# Prolonged Protection of Copper in Acidic Media Through the Synergistic Effect of Fat-Soluble Vitamins

**DOI:** 10.3390/ma18225107

**Published:** 2025-11-10

**Authors:** Regina Fuchs-Godec

**Affiliations:** Faculty of Chemistry and Chemical Engineering, University of Maribor, 2000 Maribor, Slovenia; regina.fuchs@um.si

**Keywords:** copper, fat soluble vitamins, inhibitors, EIS, long-term experiment

## Abstract

The long-term corrosion protection of copper surfaces modified with self-assembled hydrophobic layers (SAHLs) based on stearic acid (SA) and two fat-soluble vitamins, vitamin K_3_ (menadione) and vitamin E (E307), was investigated in simulated acidic urban rain (pH 5) over 7 days. The SAHLs were characterised by SEM, contact angle goniometry, ATR-FTIR, potentiodynamic polarisation, and electrochemical impedance spectroscopy (EIS). Surface modification was achieved by immersing copper samples in ethanolic SA solutions containing 2.0 wt% of fat-soluble vitamins. Variants included individual additives, (SA + 2.0 wt% K_3_) and (SA + 2.0 wt% E307), as well as mixtures with a constant total additive content of 2.0 wt%: (SA + [1.5 wt% K_3_ + 0.5 wt% E307]) and (SA + [1.0 wt% K_3_ + 1.0 wt% E307]). The (SA + 2.0 wt% K_3_) modification produced needle-like microstructures with strong short-term inhibition but poor long-term stability, while (SA + 2.0 wt% E307) formed smoother, more stable films. The mixture containing equal mass fractions of vitamins, (SA + [1.0 wt% K_3_ + 1.0 wt% E307]), exhibited a synergistic effect, yielding hierarchically structured, flower-like morphologies with high polarisation resistance and stable impedance over 7 days. These results show that combining K_3_ and E307 with stearic acid provides robust, environmentally friendly, and durable protection for copper surfaces.

## 1. Introduction

Corrosion is a major cause of many environmental disasters, both historically and in current industry. It remains a significant challenge for various industries and scientific fields, driving ongoing research and development of materials, coatings, inhibitors, and protection techniques. Fundamentally, metal corrosion results from spontaneous electrochemical reactions between the metal surface and its environment. Factors such as humidity, oxygen, chloride ions, pollutants, and temperature fluctuations accelerate these processes, leading to the gradual deterioration of metallic materials. Although considerable progress has been made in slowing corrosion, its complete elimination remains unresolved. Corrosion, as a process of material degradation, occurs wherever buildings [1,2] and industrial infrastructures are constructed from conventional materials or metallic components [3,4]. Despite the special properties of these materials (mechanical strength, durability, thermal stability, and ease of fabrication, making them suitable for engineering applications), their usefulness is still limited. One of the main limitations is corrosion and the associated material damage, which almost always accompanies corrosion reactions. Damage to industrial plants can have catastrophic consequences, both economically and in terms of irreversible environmental harm. Previous generations of corrosion inhibitors often contained heavy metals such as chromium, lead, or cadmium, now recognised as toxic and environmentally persistent. Although their use has largely been phased out due to strict environmental regulations, they are still occasionally present in certain industrial formulations. This highlights the importance of developing sustainable and environmentally friendly corrosion protection systems based on non-toxic, biodegradable components. The consequences for the environment [2,5], such as leaks and pollution, further emphasise the importance of sustainable corrosion protection methods. Additionally, the core principles of the circular economy encourage the search for potential raw materials in waste [6,7].

Corrosion inhibitors are among these potential resources (e.g., agro-industrial by-products such as polyphenols, flavonoids, and tannins from fruit peels or grape pomace, bio-based extracts from seeds and other plant materials, residues of commercial inhibitors such as discarded or outdated products based on amines, azoles, or phosphates, and leftover compounds from coatings or partially used cleaning agents, e.g., benzotriazole derivatives from cooling fluids, expired food supplements). Among these expired supplements are also various vitamins, which are already known to be promising green inhibitors. Numerous studies suggest that water-soluble vitamins—such as vitamin C [8,9,10,11,12,13,14,15,16], vitamin D, vitamin E [17,18,19,20,21] and the B-complex vitamins (including thiamine (B1) [9,22], riboflavin (B2) [16], niacin (B3) [8,23], pantothenic acid (B5) [24], vitamin B6 [8,16], biotin (B7) [22], folic acid (B9) [25] and vitamin (B12) [26]—also act as effective corrosion inhibitors. Modern inhibitors not only protect materials from the destructive effects of corrosion but also increase the functional value of the protected surface by modifying its original physical properties. These surfaces can become self-cleaning and are resistant to moisture due to the hydrophobic properties they develop—they do not thaw or freeze. These properties are also very effective in preventing corrosion [27,28,29,30,31,32].

Self-assembled hydrophobic layers (SAHLs) represent an advanced and sustainable strategy for the corrosion protection of metal surfaces. These nanometer-thick layers form spontaneously through the chemisorption of functional organic molecules on metal substrates. The molecules usually contain a head group—such as thiols, silanes, phosphonates or carboxylic acids which binds strongly to the metal surface, and a hydrophobic tail, which often consists of long-chain aliphatic groups such as fatty acids and forms a dense, water-repellent barrier [18,19,20,21,33,34,35,36,37]. Structurally, vitamins mentioned above contain polar head groups, such as hydroxyl (–OH) (vitamins C, E, B2, B6), carbonyl or carboxyl (C=O, –COOH) (vitamins C, B3, B5, B9), amine or amide (–NH_2_, –CONH–) (vitamins B1, B6, B9, B12), and sulphur-containing groups (–S–) (vitamins B1, B7), which can strongly adsorb onto copper surfaces through coordination or hydrogen bonding. The remaining nonpolar or aromatic moieties—for example, the alkyl tail in vitamin E or the conjugated rings in B2, B6, and B9—act as hydrophobic tails, forming a protective barrier that limits the access of corrosive species to the metal. This dual structural nature explains their efficiency as green corrosion inhibitors. Fatty acids have proven to be particularly effective components in SAHL formation due to their natural occurrence, biodegradability, and strong affinity for metal oxides. Their hydrophobic character reduces surface wettability, limiting moisture absorption and slowing electrochemical corrosion processes. SAHLs based on fatty acids and related compounds not only form a physical barrier that hinders the diffusion of oxygen, chloride ions, and other aggressive species, but are also ultrathin, nearly invisible layer that does not alter the physical properties of the substrate, such as surface roughness, hardness, and visual appearance. In addition, the molecular structure of SAHLs can be customised to provide specific functionalities. For example, self-healing behaviour can be achieved by encapsulating active inhibitors in smart coatings, antibacterial properties result from materials that inhibit microbial growth, and improved adhesion can be achieved through surface microtexturing [27,28,38]. Compared to conventional protection methods, which often use toxic or environmentally harmful coatings and preservatives (such as metal-based paints, solvent-based polymers, or chemical biocides) to prevent corrosion, microbial growth, or moisture damage, these SAHLs are non-toxic, environmentally friendly, and conform to green chemistry principles, making them ideal for applications in the marine, electronics, and food industries where mechanical stress is low.

In our previous studies [19,20,21,22], SAHLs were formed using fatty acids as the primary structural component, taking advantage of their strong affinity to metal oxide surfaces and their excellent water-repellent properties. To improve the corrosion resistance of these layers further, α-tocopherol (vitamin E, E307), a fat-soluble antioxidant, was integrated into the system. These SAHLs exhibited an inhibition efficiency of up to 99% (*η* = 99%) against metal corrosion in various systems, which has encouraged us to expand our research further [18,19,20,21]. Specifically, we investigated the inhibitory effect of self-assembling monolayers (SAHLs) formed on pure copper and copper alloy in simulated urban rain with pH 5 [18], and on stainless steel (SS) type AISI 410S in 3.0 wt% NaCl [21], as well as the long-term stability and effectiveness of the SAHLs on SS AISI 410S in 3.0 wt% NaCl [20] and in simulated urban rain with pH 3 at 25 °C [19]. The most effective inhibition in the selected corrosion media was observed for SAHLs formed on metal surfaces in an ethanol solution containing 0.05 M SA with the addition of E307 at a concentration of 2.0 wt%. Therefore, in this study, the concentration of the added vitamin was maintained at 2.0 wt%, whether added individually or in combination.

The focus of this study was on three aspects: (i) evaluating the durability of these specific SAHLs on the copper surface in long-term tests, up to 7 days after immersion in a selected corrosive medium (urban acid rain, pH = 5); (ii) extending the approach to another fat-soluble vitamin, vitamin K_3_ (2-methyl-1,4-naphthoquinone=menadione), using the same procedure for SAHLs formation as reported in our previous studies [18,19,20,21]; and (iii) exploring the potential synergistic effect of a combination of inhibitors (K_3_ + E307), as several studies suggest that mixtures of inhibitors may provide better protection than single-component systems. Two mixed compositions of added vitamins were tested in the solution used for copper surface modification, with a total vitamin concentration of 2.0 wt%. The first solution contained (SA + [1.5 wt% K_3_ + 0.5 wt% E307]), while the second contained (SA + [1.0 wt% K_3_ + 1.0 wt% E307]), both prepared in an ethanolic solution of stearic acid. While previous studies demonstrated effective corrosion inhibition of SAHLs with E307, the long-term durability on copper and the potential use of other fat-soluble vitamins remained unexplored. In this work, SAHLs formed with vitamin K_3_ were investigated; these showed good inhibition after one day but a significant decrease after four days, whereas E307 provided maximal protection after four days. This led to the development of mixed systems combining K_3_ and E307, aiming to unite the positive temporal effects of both vitamins. By evaluating these multi-component layers over extended immersion times, this work addresses both the novelty of multi-vitamin SAHLs on copper and their potential as long-term, stable, and sustainable corrosion protection systems, providing insights into the design of environmentally friendly and effective corrosion inhibitors. The morphology of the copper surfaces modified with SAHLs was characterised by scanning electron microscopy (SEM), while the wettability of the copper surfaces with and without SAHLs was evaluated using a commercially available goniometer (OCA25). Attenuated total reflection Fourier transform infrared spectroscopy (ATR-FTIR) was used to gain insight into the possible corrosion protection mechanisms. The long-term corrosion resistance of the SAHLs was evaluated using two electrochemical techniques: classical potentiodynamic measurements (PDM) and electrochemical impedance spectroscopy (EIS). Based on these results, the corrosion protection performance of the SAHLs over extended exposure periods is discussed in detail.

## 2. Materials and Methods

### 2.1. Materials and Chemicals

**Scheme 1:**
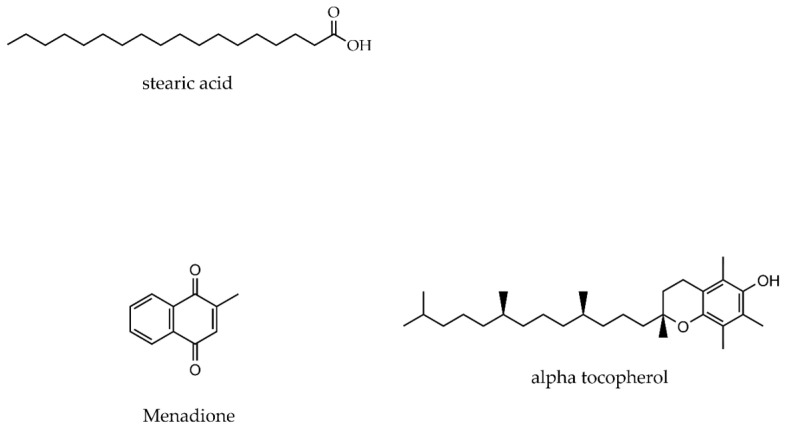
Structural formulae of the component used in this study.

Cylindrically shaped specimens were made from pure copper (99.95% purity). The following chemicals were used to prepare the test solution, i.e., the simulated acid rain, NaNO_3_ (Merck GesmbH, Graz, Austria, CAS number 7631-99-4), NaHCO_3_, (J.T. Baker, Phillipsburg, NJ, USA, CAS 144-55-8) and Na_2_SO_4_ (Merck GesmbH, Graz, Austria, CAS number 7757-82-6). The solution itself was acidified to pH 5 with H_2_SO_4_. The stearic acid used (Sigma-Aldrich, Darmstadt, Germany CAS number 57-11-4, St. Louis, MO, USA) was of pure grade (95%), while the (+)-α-tocopherol (vitamin E, E307) was also a pure product (97%) from Sigma-Aldrich, Darmstadt, Germany (CAS number 59-02-9) and, Menandion (≥98%) from Carl Roth GmbH + Co.KG, Karlsruhe, Germany (CAS No. 58-27-5). All the chemicals were used as received and without further processing. The structural formulas of the components used in this study are shown in the Scheme 1.

### 2.2. Instrumentation

The present investigation includes measurements on the following apparatus:

Electrochemical measurements:

– Gamry 600™ potentiostat/galvanostat, Warminster, PA, USA, controlled by an electrochemical program.
– Processing and analysis of experimental data using the following software programs: CorrView2, CorrWare, Zplot and ZView4 programs from Scribner Associates, Southern Pines, NC, USA (all version 2.80).
– ATR-FTIR: SHIMADZU-IRAffinity-1, Shimadzu Europa GmbH, Duisburg, Germany.
– Scanning Electron Microscope (SEM): JSM IT800SHL; Joel, Tokyo, Japan.
– Goniometer: Data Physics OCA 25, Filderstadt, Germany.

### 2.3. Pretreatment for the Electrode and Electrochemical Measurements

**Scheme 2:**
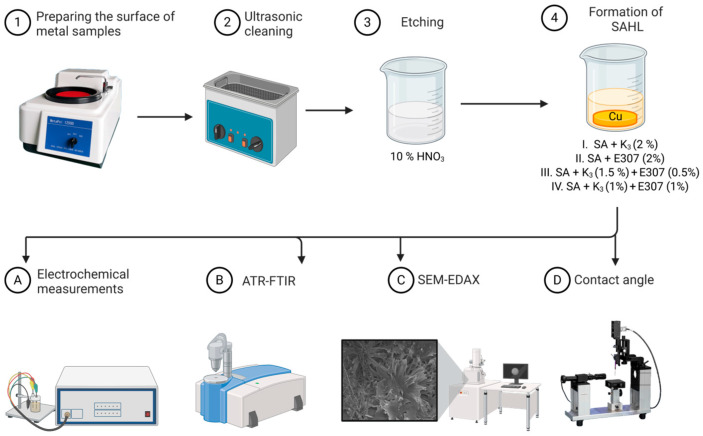
Schematic diagram of sample preparation and fabrication of the SAHLs, as well as the techniques used for measurements and further analyses. The scheme was made in Biorender (https://biorender.com/, accessed date: 10 September 2025).

Prior to etching, the metal substrates were polished mechanically using a flow-assisted abrasion setup with silicon carbide (SiC) papers of progressively finer grit sizes (800, 1000 and 1200), under running water to minimise contamination. After grinding, the samples were cleaned in an ultrasonic bath to remove any grinding residue. The surface was then etched in a 10% aqueous HNO_3_ solution for 20 s and then rinsed with deionised water. The samples were then subjected to ultrasonic cleaning again in Milli-Q water, washed with ethanol, rinsed thoroughly with distilled water and dried with compressed air. The cleaned copper substrates were then immersed at room temperature for approximately one hour in a 0.05 mol/L ethanolic solution of stearic acid (SA) containing a selected mass fraction of added K_3_, E307, or their combination: (a) (SA + 2.0 wt% E307), (b) (SA + 2.0 wt% K_3_), (c) (SA + [1.5 wt% K_3_ + 0.5 wt% E307]), and (d) (SA + [1.0 wt% K_3_ + 1.0 wt% E307]) to form hydrophobic layers based on stearic acid. In the following text, “wt%” is omitted when indicating the weight concentration of K_3_ or E307, and only percentage values are given. The electrochemical characterisation was carried out using potentiodynamic polarisation in a standard three-electrode cell. A saturated calomel electrode (SCE) served as the reference, a platinum wire as the counter electrode. The potential of the working electrode was sampled from −0.3 V to 0.3 V against the SCE at a scan-rate of 1 mVs^−1^. The SCE was connected to the system via a Luggin capillary placed in close proximity to the working electrode to minimise IR drop. The impedance spectra were determined at constant open-circuit potential (OCP) in the frequency range from 100 kHz to 1 mHz, with 10 points per decade and an amplitude of 10 mV (peak-to-peak) of the excitation signal. The impedance (Nyquis and Bode) and polarisation plots were generated from the results of these experiments one hour after immersion of the working electrode in the corrosion media, to allow stabilisation of the steady-state potential (three replicate measurements were performed). All the experiments were performed at a controlled temperature of 25 °C ± 1 °C. The measurements were performed using a Gamry 600™ potentiostat/galvanostat controlled by an electrochemical programme. Electrochemical potentiodynamic measurements were performed after 1, 4 and 6 days of immersion in the acidic urban rain solutions with a pH of 5.0, and in the case of the EIS measurements after one hour, 1d, 4d, 6d and after seven days of immersion. The composition of the simulated form of acid rain: 0.2 g/L Na_2_SO_4_, 0.2 g/L NaHCO_3_, and 0.2 g/L NaNO_3_, acidified to pH = 5 with H_2_SO_4_. Before each simple measurement (EIS or potentiodynamic measurements), the OCP of the working electrode was measured for one hour to allow stabilisation of the steady-state potential. The complete sample preparation procedure and the apparatus used for measurements and further analyses are illustrated in the Scheme 2.

#### 2.3.1. Contact Angle

Contact angle (CA) measurements were performed with a Data Physics OCA 25 goniometer (Filderstadt, Germany) in a temperature-controlled environment at 25 ± 1 °C. A drop of ultrapure water (1 µL) was applied carefully to modified and unmodified copper substrates. Five measurements were performed for each surface type, and the mean contact angle value was reported.

#### 2.3.2. ATR-FTIR Analysis

Attenuated total reflection Fourier transform infrared spectroscopy (ATR-FTIR) was used to determine the chemical composition of the self-assembled hydrophobic coating. The obtained spectra were compared with reference vibrational band positions to identify functional groups. The measurements were performed on copper surfaces modified with SAHLs: (SA + 2.0 E307), (SA + 2.0 K_3_), and (SA + [1.0 K_3_ + 1.0 E307]). The spectra were recorded with a resolution of 4 cm^−1^ over the wavenumber range of 400–4000 cm^−1^.

#### 2.3.3. Surface Characterisation Using SEM/EDX

The surface morphology of the self-assembled coatings was examined using a scanning electron microscope (SEM, XL FEG/SFEG/SIRION) equipped with an energy-dispersive X-ray spectroscopy detector (EDX). The imaging was conducted at accelerating voltages of 15 and 20 kV to ensure proper excitation of all the detectable elements.

## 3. Results and Discussion

### 3.1. Wettability of a High-Level Hydrophobic Surface

**Figure 1:**
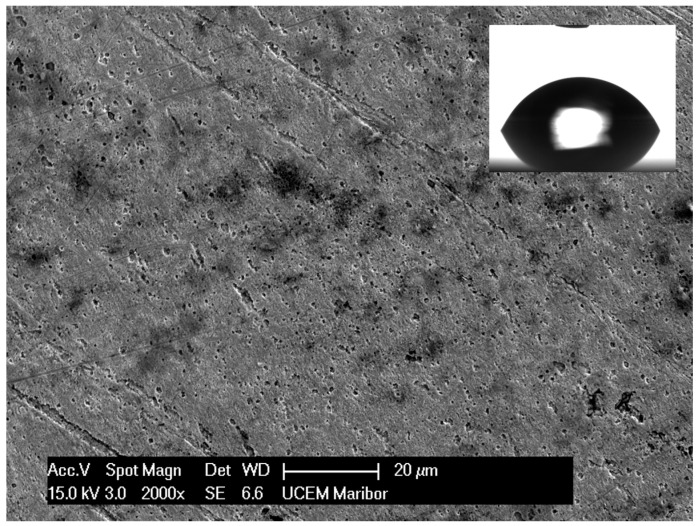
SEM of the untreated surface of Cu with CA = (64.0° ± 2) [18].

**Figure 2:**
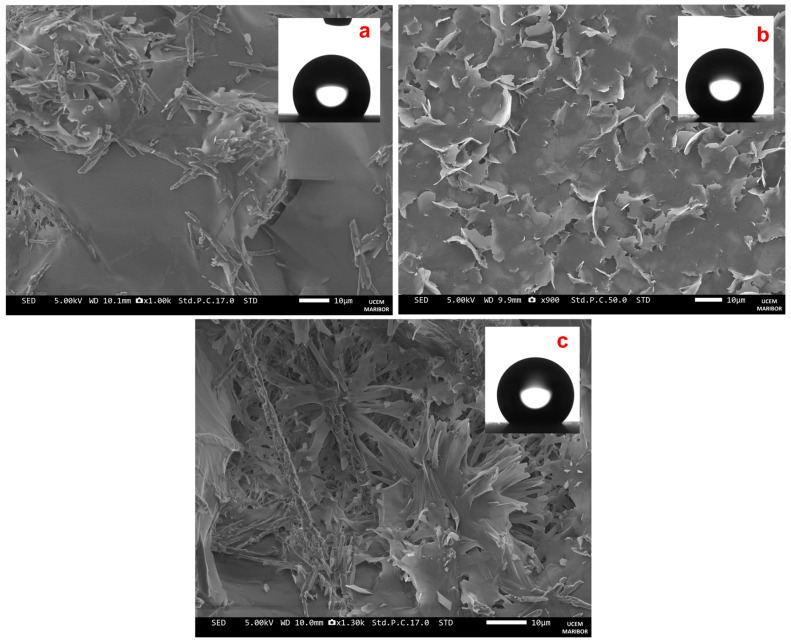
SEM images of Cu samples treated in ethanol solution SA for one hour with various additions of vitamins K_3_ and/or E307; (**a**) (SA + 2.0% K_3_), CA = (122.74° ± 1) (**b**) (SA + 2.0% E307), CA = (140.24° ± 1), and (**c**) (SA + [1.0% K_3_ + 1.0% E307]), CA = (137.32° ± 1).

The evaluation of the corrosion protection properties of the hydrophobic surfaces was carried out mainly by contact angle measurements, the investigation of the surface morphology of both modified and unmodified copper by means of SEM and electrochemical measurements in a solution of acidic urban rain [18,19,20,21]. Figure 1 and Figure 2 show the surface microstructures of the bare (Figure 1) and hydrophobic copper samples as examined by SEM. The images in Figure 1 and Figure 2 show the profiles of water droplets on the bare and modified surfaces. They show that the hydrophobicity of the modified surfaces with CA values are not too different from those of the superhydrophobic surfaces (CA > 150°) [39,40,41,42,43,44]. Figure 1 shows the SEM image of the untreated copper surface, with a measured contact angle of 64.0°. The contact angle confirms the hydrophilic nature of the copper surface. SEM analysis of the (SA + 2.0% K_3_) composites shows needle-like structures with dimensions of several hundred nm (Figure 2a). In contrast, the surfaces modified with (SA + 2.0% E307) (Figure 2b) or with the vitamin mixture (SA + [1.0% K_3_ + 1.0% E307]). (Figure 2c) showed a hierarchically organised architecture. The mixed system exhibited a distinct flower-like morphology, characterised by faceted, crystal-like domains—presumably associated with K_3_—interspersed with smoother, amorphous-like regions attributable to E307. This biphasic, interwoven structure produced multiscale surface features, ranging from micrometre-scale aggregates to nanoscale textures, promoting a hierarchical organisation. The observed surface morphology correlates strongly with the wettability. The SAHL of (SA + 2.0% K_3_) exhibited moderate hydrophobicity (CA = 122.74°), due probably to the molecular structure of K_3_ and the relatively loose needles arrangement. In comparison, the (SA + 2.0% E307) forms smoother, more uniformed layers, resulting in higher hydrophobicity (CA = 140.24°). Interestingly, the SAHL formed with the mixture (SA + [1.0% K_3_ + 1.0% E307]) retained a high contact angle (CA = 137.32°), comparable to that of (SA + 2.0% E307). This indicates that the hierarchical surface, which combines the needle structures characteristic of the (SA + 2.0% K_3_) layer with E307-rich regions, retains strong hydrophobicity and increased structural complexity. SEM analysis confirmed the coexistence of these features, revealing a multiscale morphology. These results highlight the crucial role of surface morphology in controlling wettability, demonstrating that the combination of K_3_ and E307 produces a robust, hierarchically structured hydrophobic layer on copper surfaces.

### 3.2. Corrosion Resistance of SAHLs

#### 3.2.1. Potentiodynamic Measurements (PDM)

**Figure 3:**
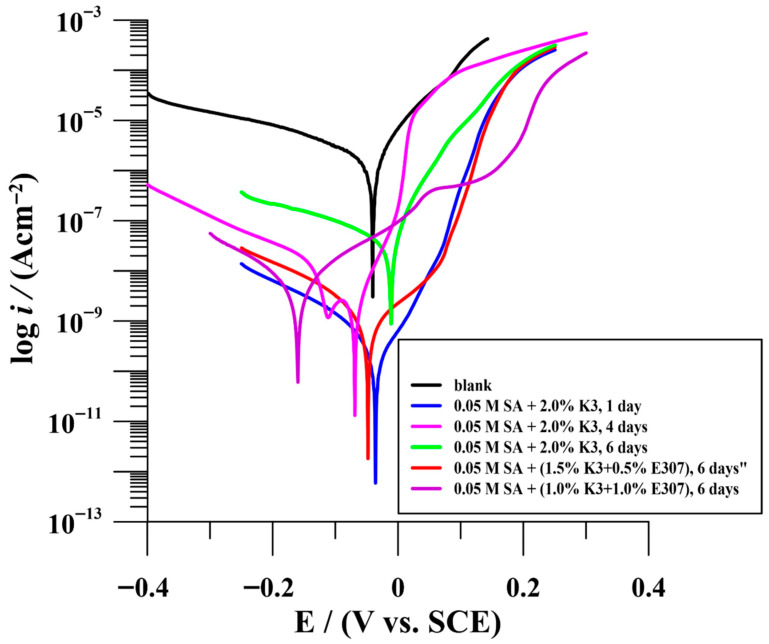
Potentiodynamic polarisation curves (1 mVs^−1^) for bare and modified copper surfaces within the acidic urban rain solutions with a pH of 5.0 at 25 °C (measured after 1, 4, and 6 days of immersion in the corrosion medium). The modified surfaces were prepared by the immersion of Cu in 0.05 M SA in ethanol with and without the addition of 2% Vitamin K_3_ or mixtures of both vitamins.

**Figure 4:**
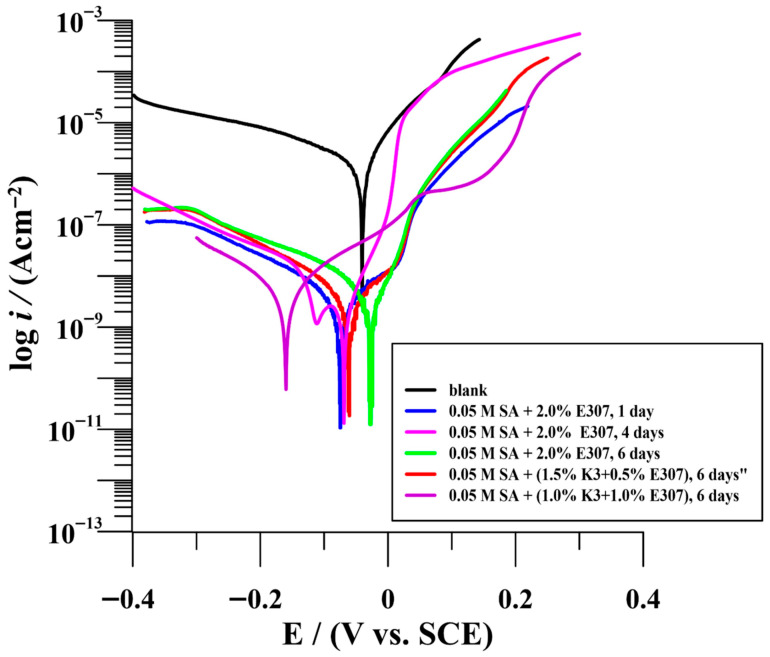
Potentiodynamic polarisation curves (1 mVs^−1^) for bare and modified copper surfaces within the acidic urban rain solutions with a pH of 5.0 at 25 °C (measured after 1, 4, and 6 days of immersion in the corrosion medium). The modified surfaces were prepared by the immersion of Cu in 0.05 M SA in ethanol with and without the addition of 2% Vitamin E307 or mixtures of both vitamins.

The potentiodynamic curves for the “acid rain” solution for bare Cu and for the surfaces modified by immersion in SA with the addition of vitamin K_3_ and E307, when used individually and in the case of a combination of both, are shown in Figure 3 and Figure 4. The measurements of the modified Cu surfaces were performed after 1, 4 and 7 days of immersion in the selected corrosion media. In the case of the mixtures of both vitamins, only a six-day exposure is shown, as we were interested primarily in the long-term stability of the hydrophobic surfaces produced in this way. Compared to the bare Cu, the modified Cu surfaces showed a decrease in current densities in both directions (cathodic and anodic). In addition, the reduction in corrosion current density *i*_corr_, was up to a fourth order of magnitude lower in the case of SAHL of (SA + 2.0% K_3_) compared to the blank solution, and remained stable for four days after sample immersion. After six days of immersion, the *i*_corr_ had already increased by two orders of magnitude, at the same time the corrosion potential *E*_corr_ shifted towards the more positive values and the inhibition effect had dropped to 97%. The increase in *i*_corr_ in combination with the anodic shift of *E*_corr_ indicates an early failure of the protective layer (SAHL). The SAHL had not yet lost its functionality completely due to the still high inhibition efficiency *η* of 97%, but it cannot be excluded that local corrosion microprocesses were already present, e.g., enlargement of the micropores, partial desorption of the stearic acid and K_3_, which could be the reason for the degradation of the SAHL (Figure 3). In the case of the SAHL of (SA + 2.0% E307), the reduction in corrosion current was up to three orders of magnitude compared to the untreated Cu surface. Although the corrosion current density during the one-to-four-day of immersion in the selected corrosion medium was slightly higher compared to the previously presented SAHLs, the SAHL of (SA + 2.0% E307), exhibited much higher stability, while the value of *i*_corr_ did not change significantly, even after 6 days of immersion, not more than about half an order of magnitude, as shown in Figure 4. The higher hydrophobicity of these SAHLs, to which the hydrophobic E307 contributes, prevents the penetration of electrolytes more effectively, which is crucial to avoid the swelling and stresses that can lead to cracks within the SAHLs. To improve the long-term inhibitory performance of the SAHL prepared with (SA + 2.0% K_3_), the copper surfaces were modified by immersing the samples in an ethanolic solution of 0.05 M stearic acid containing both previously studied fat-soluble vitamins. In the mixtures, the total concentration of added vitamins was maintained at 2.0%, split between the two selected vitamins, namely (SA + [1.5% K_3_ + 0.5% E307]), and (SA + [1.0% K_3_ +1.0% E307]), with the aim of potentially gaining insight into possible synergistic effects between the two vitamins. Figure 3 and Figure 4 also show the polarisation curves of the modified copper surface in the mixtures (SA + [1.5% K_3_ + 0.5% E307]), and (SA + [1.0% K_3_ + 1.0% E307]) after 6 days of immersion. After six days of immersion, compared to (SA + 2.0% K_3_), the SAHL (SA + [1.5% K_3_ + 0.5% E307]) exhibited a cathodic shift in *E*_corr_ of approximately 80 mV and a reduction in corrosion current density by about one order of magnitude. Increasing the E307 content to 1% in (SA + [1.0% K_3_ + 1.0% E307]) resulted in a further cathodic shift in *E*_corr_ of about 180 mV, while *i*_corr_ remained nearly unchanged. However, the anodic current density decreased across the entire anodic range, and increased much more slowly compared to SAHL (SA + [1.5% K_3_ + 0.5% E307]). Considering the known fact that copper corrosion is driven primarily by oxidation of the copper surface starting with the anodic reaction, the shift of *E*_corr_ to the cathodic region indicates a decrease in anodic activity [45,46,47]. This is due mainly to the fact that the combination of stearic acid, vitamin K_3_ and E307 forms a denser hydrophobic layer that effectively prevents the penetration of water with corrosive ions and oxygen into the copper surface as is the case with SAHL from (SA + 2.0% K_3_). SAHL with a composition of (SA + [1.5% K_3_ + 0.5% E307]) has a lower quality compared to SAHL (SA + 2.0% E307), which is particularly pronounced in the anodic range. The polarisation curve shows clearly that the anodic current density begins to rise sharply immediately at the transition from the cathodic to the anodic range. This phenomenon can be attributed to the unsuitable ratio of K_3_ to E307 in the mixture. In this case, the lack of strength of the hydrophobic properties of SAHL is clearly emphasised due to the lower concentration of E307. The improvement of the anti-corrosion properties of SAHL was achieved by increasing the content of E307 to 1.0%; (SA + [1.0% K_3_ + 1.0% E307]). In comparison with the SAHL of (SA + 2.0% E307) there was a clear shift of the *E*_corr_ into the cathodic range and a reduction in the anodic current density over the entire measured anodic range. However, based on the potentiodynamic measurements, it can be concluded that the addition of both fat-soluble vitamins in appropriate concentrations each possessing distinct corrosion-inhibiting properties led to a certain degree of optimisation of the SAHLs, particularly in terms of long-term stability. The following section (EIS) explains in more detail what has changed compared to the two previous SAHLs in which the vitamins were not added at the SAHLe time.

The electrochemical parameters obtained from these polarisation curves, the corrosion potential (*E*_corr_), the corrosion current density (*i*_corr_), the Tafel constants (*b*_a_, *b*_c_), the polarisation resistance (*R*_p_) and the inhibition effect are listed in Table 1. The polarisation resistance was determined by linear polarisation within the potential range of ±15 mV with respect to *E*_corr_. The extrapolation of the Tafel line allowed the calculation of the corrosion current density *i*_corr_. All the parameters were determined simultaneously using the CorrView2 software.

#### 3.2.2. EIS Measurements

**Figure 5:**
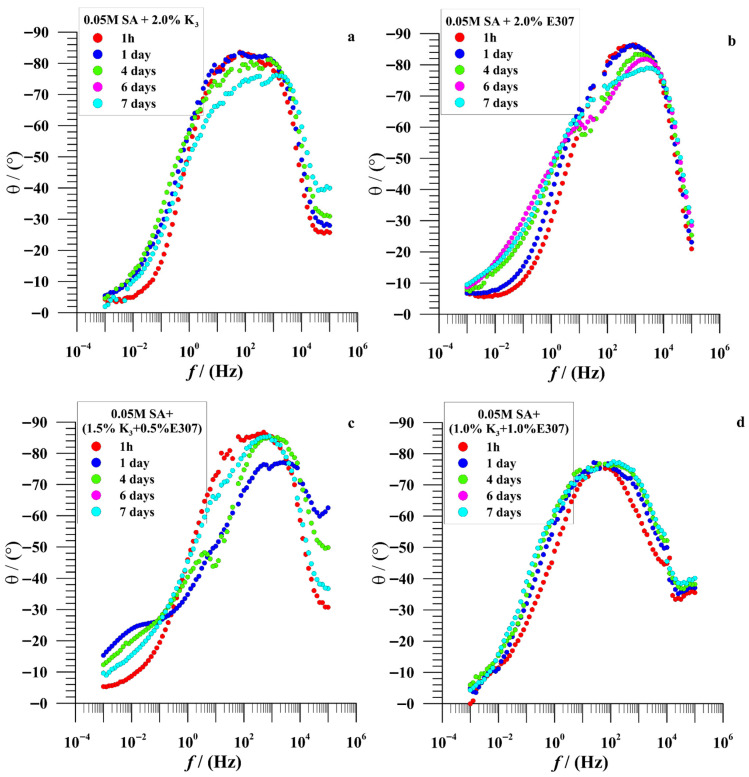
Bode-phase angle plots for copper samples for bare and modified surfaces in urban acid rain (pH = 5) measured after 1 h, 1, 4, 6 and 7 days of immersion at its open-circuit potential. (The modified surfaces were prepared by the immersion of the Cu in 0.05 M SA in ethanol with and without the addition of 2% Vitamin K_3_, Vitamin E or mixtures of both vitamins). (**a**) modified surface with (SA + 2.0% K_3_), (**b**) modified surface with (SA + 2.0% E307), (**c**) modified surface with (SA + [1.5% K_3_ + 0.5% E307]), (**d**) modified surface with (SA + [1.0% K_3_ + 1.0% E307]).

**Figure 6:**
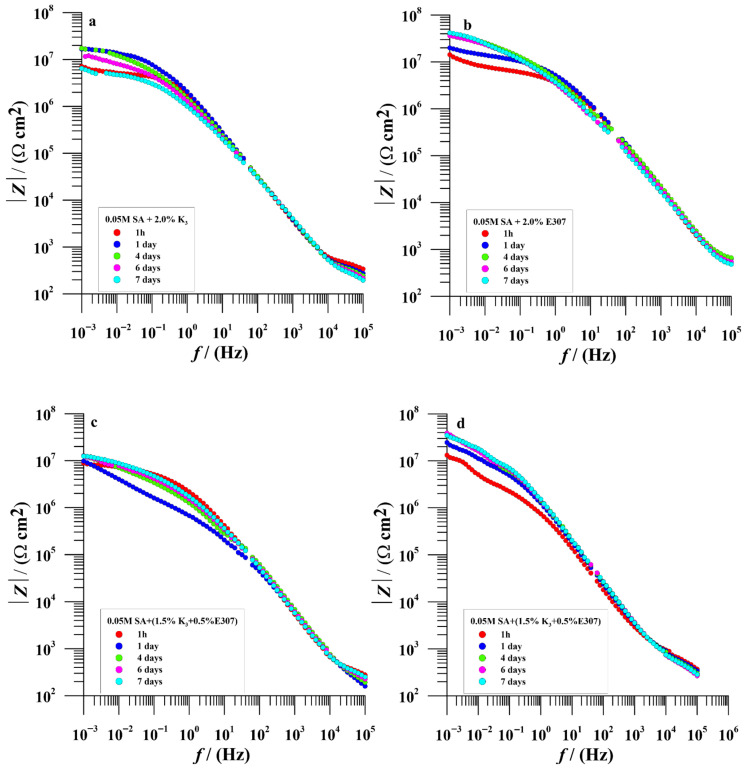
Bode-|Z|-module for copper samples for bare and modified surfaces in urban acid rain (pH = 5) measured after 1 h, 1, 4, 6 and 7 days of immersion at its open-circuit potential. (The modified surfaces were prepared by the immersion of the Cu in 0.05 M SA in ethanol with and without the addition of 2% Vitamin K_3_, Vitamin E or mixtures of both vitamins). (**a**) modified surface with (SA + 2.0% K_3_), (**b**) modified surface with (SA + 2.0% E307), (**c**) modified surface with (SA + [1.5% K_3_ + 0.5% E307]), (**d**) modified surface with (SA + [1.0% K_3_ + 1.0% E307]).

**Figure 7:**
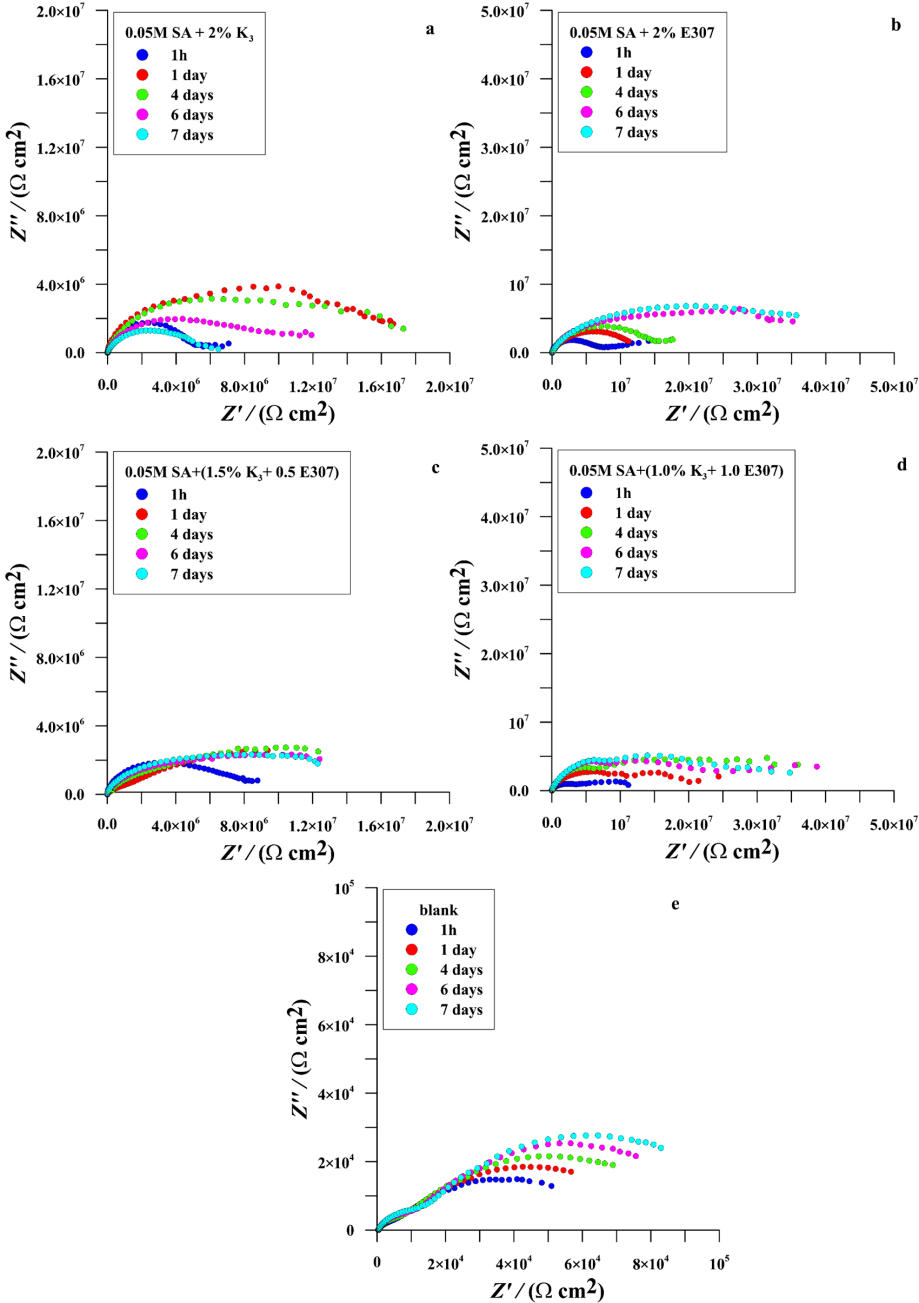
EIS Nyquist plots for copper samples for bare and modified surfaces in urban acid rain (pH = 5) measured after 1 h, 1, 4, 6 and 7 days of immersion at its open-circuit potential. (The modified surfaces were prepared by the immersion of the Cu in 0.05 M SA in ethanol with and without the addition of 2% Vitamin K_3_, Vitamin E or mixtures of both vitamins). (**a**) modified surface with (SA + 2.0% K_3_), (**b**) modified surface with (SA + 2.0% E307), (**c**) modified surface with (SA + [1.5% K_3_ + 0.5% E307]), (**d**) modified surface with (SA + [1.0% K_3_ + 1.0% E307]), (**e**) bare surface.

**Figure 8:**
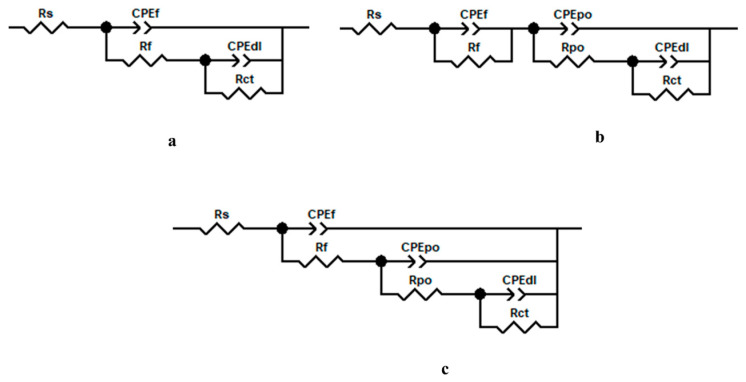
Equivalent circuits for EIS results for the bare and as-prepared hydrophobic surfaces of copper samples. (**a**) bare surface, (**b**) SAHLs (SA + 2.0% K_3_), (**c**) SAHLs (SA + 2.0% E307), (SA + [1.5% K_3_ + 0.5% E307]) and (SA + [1.0% K_3_ + 1.0% E307]).

Electrochemical impedance spectroscopy (EIS) is nowadays an extremely useful method for evaluating the corrosion resistance of protective layers (e.g., oxides), paints or coatings on metals, etc. Its main advantage is that it enables non-invasive monitoring of the corrosion process in real time without damaging the protective layer(s) or the metal surface [48,49,50,51,52,53]. Based on the EIS measurements and further considerations with the introduction of an appropriate electrical equivalent circuit EEC, the important parameters related to the condition of the protective coating on the metal surface can be determined, i.e., the resistance of the protective layer (higher impedance in the low frequency range indicates better protection), the capacitance of the protective layer—changes in the value (increasing mode) indicate a possible penetration of water or electrolyte into the interior of the protective layer (coating, paint). In addition, the charge transfer resistance is another important parameter that is crucial for evaluating the rate of the corrosion process at the coating/metal interface and the diffusion processes that normally occur, especially if the protective layers are exposed to the corrosive medium over a longer period of time. In electrochemical impedance spectroscopy (EIS), the Nyquist and Bode diagrams, together with a suitable electrical equivalent circuit and its elements, are the most important instruments for evaluating the state of the sample under investigation [54,55,56]. The Bode diagrams from our study are shown in Figure 5a–d and Figure 6a–d, while the Nyquist diagrams are presented in Figure 7a–e. The most suitable electrical equivalent circuits (EECs) used are shown in Figure 8a–c.

Figure 5a–d and Figure 6a–d show the Bode diagrams of the modified Cu surfaces after various immersion times in a naturally aerated, simulated acidic urban rain solution. The Bode plots illustrate the changes in phase angle and impedance modulus (|Z|) as functions of frequency on a logarithmic scale. When an organic coating such as SAHL is applied to a metal surface, the impedance response is typically observed across the high-frequency (HF), mid-frequency (MF), and low-frequency (LF) regions and provides valuable information about various processes within the coating and at the interface. Figure 5a–d show a wide phase angle for all four SAHLs on the Cu surface, indicating the presence of several overlapping time constants, two in the case of the bare copper surface (2 RC elements) and three in the case of the SAHLs (3-RC elements). This is reflected in the composition of the electrochemical equivalent circuit images (EECs) used to fit the experimental data. Figure 8 shows the EECs used, which provided the fitting curves that match the experimental data best. In the Bode plot obtained for the modified Cu surfaces (Figure 5a–d), the values of the phase angle of all four different SAHLs were on average between −80° and −85°, which is very close to −90° in a wide frequency range. In particular, the investigated SAHLs showed a capacitive behavior in the frequency range between 5 kHz and 5 Hz, where the phase angle reaches a maximum value, outside this range the phase angle increases or decreases, indicating that both capacitive and resistive behavior coexist in this range.

The phase angle values between −80° and −85° over a wide frequency range promised basically good corrosion protection properties of SAHLs in all four cases [57,58,59].

A Nyquist diagram shows the impedance of an electrochemical system, where the real part (resistance) is plotted on the x-axis and the negative imaginary part (capacitive or inductive behaviour) on the y-axis. This provides information about charge transfer, double layer capacity, diffusion processes and surface properties [19,48,53]. All the Nyquist diagrams obtained from our EIS measurements for all SAHLs show a depressed capacitive loop over the entire frequency range shown in Figure 7a–d. In such cases, typical EEC elements in corrosion systems consist of a combination of resistors (R) and a constant phase element (CPE). The constant phase element (CPE) is often used in electrochemical impedance spectroscopy (EIS) to account for non-ideal capacitive behaviour caused by surface roughness, microporosity, inhomogeneities or distributed time constants in the system. By including a CPE instead of an ideal capacitor in the electrical equivalent circuit (EEC), the modelled curves usually fit the experimental data better, especially when organic coatings, corrosion layers or porous surfaces are involved. To determine the capacitances from the values of the CPE, the following equation (Equation (1)) [18,19,20,21] was used, where:

(1) $$C={\left(Q\cdot R^{1-n}\right)}^{\frac{1}{n}}$$

*Q* is a proportionality coefficient, its value is the CPE value obtained in the adjustment, *n* is an exponent that refers to the phase shift and corresponds to the values of the fitting procedure. When *n* = 1, *Q* represents the pure capacitance, while for *n* < 1 it can be a sign of surface heterogeneity or further charge transfer reactions [60,61].

Further, *R*_s_ in EEC represents the resistivity of the electrolyte, *R*_f_ is the resistivity of the SAHL or oxide that forms on the unmodified copper surface (at the electrolyte/film phase boundary), *R*_po_ is the resistivity of the pores that result from the self-organisation of the protective layer, and *R*_ct_ refers to the resistivity of the charge transfer reactions that take place on the surface of the metal matrix. Then, there are the corresponding capacitances *C*_f_, the SAHL capacitance, the pore capacitance *C*_po_ and the double layer capacitance *C*_dl_. For fitting the experimental points from the EIS measurements, the electrical equivalent circuit shown in Figure 8a was used for the unmodified surface, for the matching of the system (SA + 2.0% K_3_) the circuit diagram shown in Figure 8b and for the others (SA + 2.0% E307), (SA + [1.5% K_3_ + 0.5% E307]) and (SA + [1.0% K_3_ + 1.0% E307]). Figure 8c. The values of the electrochemical impedance parameters determined by the fitting procedure are listed in Table 2. Based on the parameters determined in the fitting procedure, the differences between them in long-term measurements are explained in more detail below.

##### Bare Surface

It is known that under oxidising conditions (oxygen and moisture) copper first oxidises to Cu^+^, which is then converted rapidly to Cu^2+^, especially in the presence of an electrolyte or more precisely in an acidic medium [62,63,64]. The value of *R*_f_ (film resistance) increased from 1.5 kΩ cm^2^ to 8.8 kΩ cm^2^ during a seven-day immersion in a simulated acidic rain solution with pH = 5, which could be explained by the formation of an oxide layer of Cu_2_O on the copper at the beginning of the measurements. The further gradual increase in the resistance of the surface layer was most likely due to the formation of an oxide or passivation layer between the initially formed Cu_2_O and CuO or a combination of both [64,65]. The increasing value of *R*_f_ and the decreasing value of *C*_f_ indicate that the oxide layer becomes thicker and more resistant over time. At the same time, the decrease in the parameter *n*_f_ (from 0.86 to 0.66) indicates a more distributed capacitive behaviour, which means that the oxide layer develops an increasing structural inhomogeneity. The sharp decrease in *C*_dl_ (from 3297 μF cm^−2^ to 96 μF cm^−2^) indicates a significant reduction in the active surface area, which means that the copper surface is covered increasingly by an oxide or passive layer. Although a low and decreasing *C*_dl_ value typically reflects an improvement in the protective properties of the surface layer, the relatively low *n*_dl_ values (0.5–0.59) point to a non-ideal, diffuse capacitive behaviour. This behaviour may be related to a porous or non-uniform oxide layer, probably consisting of a Cu_2_O/CuO mixture with areas containing adsorbed water or local defects. Such features mean that the protective layer may become porous or even brittle over time, potentially allowing limited corrosion. The formation of corrosion products that appear to accumulate on the surface is supported by the observed increase in *R*_p_ (polarisation resistance) with prolonged immersion shown in Figure 7e. Polarization resistance (*R*_p_), representing the overall corrosion resistance, corresponds to the sum of *R*_f_ and *R*_ct_ in spontaneously passivated specimens, whereas in specimens modified with a SAHL it is expressed as *R*_f_ + *R*_po_ + *R*_ct_ [66].

##### Copper Surface Modified with (SA + 2.0% K_3_)

In the case of the copper surface modified with SAHL with the composition of (SA + 2.0% K_3_), the maximum diameter of the depressed semicircle shown in the Nyquist diagram (Figure 7a) developed after the first day of immersion, and polarisation resistance reached value of *R*_p_ = 17.9 MΩ cm^2^. After four days it began to decrease noticeably and droped to *R*_p_ = 6.5 MΩ cm^2^ after 7 days. The SAHL response can also be explained by the chemical structure of SAHL, which consists of K_3_ and SA, as well as by the results of the fitting procedure and the Bode diagrams.

The K_3_ molecule (menadione) contains a carbonyl group (C=O) that can donate its electron pairs to form a coordination bond with the copper ions on the surface. The coordination bond between the vitamin K_3_ molecule and the copper ions on the surface is formed when the copper surface is oxidised or activated, i.e., when copper ions (Cu^2+^ or Cu^+^) are present on the surface. The oxygen atom of the carbonyl group donates its unbound electron pairs to the copper ion. This creates a stable bond between the vitamin K_3_ and the copper surface, which contributes to the strong adhesion of the SAHL. At the same time, SA binds directly to the copper surface with its polar part, i.e., with the orientation of the carboxyl groups towards the metal, while its long alkane chains are oriented towards the solution and form a hydrophobic layer, which facilitates inhibition further [67,68,69,70].

The high value of *n*_f_ (0.998) indicates a homogeneous structure of the outer layer without major defects, apart from a slight microporosity, which is unavoidable in SAHL, which was also confirmed by a constant capacitance *C*_f_ (0.01 μF/cm^−2^), indicating a stable thickness and dielectric properties of the outer layer. The decrease of *R*_f_ from 204 Ω cm^2^ to 165 Ω cm^2^ during the immersion time of up to 4 days could indicate that the penetration of the electrolyte into the SAHL starts slowly. The emphasis was on slow, because the resistance of the pores was still high and had increased from 3.9 MΩ cm^2^ to 4.0 MΩ cm^2^, the pore capacitance *C*_po_ was constant (0.06 μF/cm^−2^), as was the parameter *n*_po_, which did not change over time and was 0.94, and the charge transfer resistance *R*_ct_ had decreased slightly from 14.0 MΩ cm^2^ to 13.5 MΩ cm^2^. However, the coordination bonds alone are clearly not sufficient to ensure long-term stability, as the penetration of water and electrolytes destabilised the K_3_, allowing the SAHL to reorganise and increase its hydrophilicity, which accelerated corrosion. This was confirmed by the decrease in the *R*_f_ value to 137 Ω cm^2^ during the immersion period of 4 to 7 days, which means that the SAHL became more permeable to the electrolyte.

The carboxylate groups of the SA stearic acid in the SAHL are bound to the copper surface via Cu^2+^ carboxylate bonds. An acidic pH value can lead to partial protonation of these groups. The protonated form (R-COOH) is less polar and bound less strongly to the copper surface, as the Cu^2+^ ions no longer have a strong electrostatic interaction with the R-COOH group, which can lead to a loss of stability of the layer [71,72]. The decrease in the protective function of the SAHL due to the penetration of water and corrosive ions after four days is also evident in the decrease in *R*_po_ from 4.0 MΩ cm^2^ to 0.035 MΩ cm^2^, and in *R*_ct_ from 14.0 MΩ cm^2^ to 6.5 MΩ cm^2^ as well as in an increase in the capacitance of *C*_po_ from 0.06 μF cm^−2^ to 0.1 μF cm^−2^ and of *C*_dl_ from 5.6 μF cm^−2^ na 7.9 μF cm^−2^.

##### Copper Surface Modified with (SA + 2.0% E307)

In contrast to the SAHL formed on the basis of the mixture (SA + 2.0% K_3_), the Nyquist diagram for the surface modified on the basis of a mixture with the composition of (SA + 2.0% E307) shows an extreme increase in the diameter of the depressed semicircle (*R*_p_) after 4 days of immersion in the selected corrosion medium, from 18 MΩ cm^2^ to 41 MΩ cm^2^ (Figure 7b). This can be attributed to the process of molecular reorganisation in the SAHL itself. Obviously, although the SAHL already has pronounced inhibitory properties, it is not completely homogeneous immediately after its formation. The presence of microporosity or other minor defects makes the SAHL much more vulnerable at the interface between the layer and the corrosive medium, allowing the slow penetration of electrolyte (water and acid rain ions) into the interior, as evidenced by the decrease in the *R*_f_ value after one day of immersion up to four days. The *R*_f_ value dropped from 1700 Ω cm^2^ to 800 Ω cm^2^ and to 579 Ω cm^2^ during the 7-day immersion. The slow electrolyte penetration (low water uptake) can be confirmed by the low and constant values of the film capacitance *C*_f_ = 0.008 μF cm^−2^, which indicates the internal stability of the SAHL. Due to the composition of the SAHL the interior of the pore is hydrophobic. The alkyl tails of stearic acid are highly hydrophobic. When water or an electrolyte penetrates the micropore, the alkyl tails repel water due to hydrophobic interactions (the hydrophobic effect). The alkyl tails move closer together due to the hydrophobic effect, while the polar heads of the stearic acid (–COO^−^) remain bound to the copper surface. It can be assumed that the hydrophobic effect triggers a process of molecular reorganisation simultaneously, as the E307 molecules also have hydrophilic and hydrophobic moieties, and are redistributed with their hydrophobic moieties between the alkyl chains of stearic acid, resulting in a denser and more homogeneous layer that reduces porosity and improves compactness, enhancing the inhibitory properties of SAHL further, as confirmed by the EIS results. The values of *R*_po_ increased steadily from 6.7 MΩ cm^2^ to 13.2 MΩ cm^2^, with a remarkable increase after 4 days of immersion from 8.3 MΩ cm^2^ to 13.2 MΩ cm^2^, with an average pore capacity *C*_po_ of 0.025 μF cm^−2^. Compared to *C*_f_, *C*_po_ is around half of an order of magnitude higher, confirming the electrolyte penetration into the pores, which, at the same time, causes the above-mentioned reorganisation of the molecules due to the hydrophobic effect aimed at displacing polar water molecules from the hydrophobic areas, as evidenced by the increase in *R*_po_ after 4 days of exposure. The high pore resistance in turn contributed to the high charge transfer resistance *R*_ct_, which increased steadily from 2.3 MΩ cm^2^ to 31.5 MΩ cm^2^ during the 7-day time window and increased sharply from 10.3 MΩ cm^2^ to 31.5 MΩ cm^2^ after 4 days of immersion. It could be surmised that the water that initially entered the pores is displaced gradually by the compaction of the alkyl tails and the redistribution of the E307 molecules, which was confirmed by the decrease in the *C*_dl_ capacitance from 6.86 μF cm^−2^ to 1.08 μF cm^−2^ after 7 days of immersion. Last but not least, the polarisation resistance *R*_p_, which results from the sum of the contributions of all three resistances, increased from 9.1 MΩ cm^2^ to 41.7 MΩ cm^2^. The increased protection of the copper surface was achieved by blocking the penetration of corrosive ions from the solution.

The EIS measurements confirmed that the addition of K_3_ or E307 to stearic acid during the formation of a self-assembled hydrophobic layer (SAHL) on the copper surface improved its inhibition properties. If the SAHL consists only of SA, its inhibition effect is lower, as we have already shown in our previous studies [18,19,20,21]. The combination (SA + K_3_) has an immediate effect on increasing the inhibition effect already after 1 h of exposure, but the effect is limited to a shorter time window of up to 4 days, while the mixture (SA + E307) has an effect on the stability of the corrosion protection during a longer exposure in a simulated acid rain solution, at least up to 7 days, when SAHL is formed. Therefore, the challenge was to improve the inhibition properties of SAHL in the (SA + K_3_) combination, as the effect of K_3_ was not sufficient to protect the copper surface during prolonged exposure to the chosen corrosive environment. Considering that each of the fat-soluble vitamins was effective in different time windows, a synergistic effect could be expected when both interacted in an appropriate ratio, which could lead to even more effective protection in the long term. We limited the amount of added vitamins to 2.0% as in the first two cases, which could give an insight into the possible synergistic effect between them in the formation of SAHL depending on the chosen ratios between them.

##### Copper Surface Modified with (SA + [1.5% K_3_ + 0.5% E307]) and (SA + [1.0% K_3_ + 1.0% E307])

(SA + [1.5% K_3_ + 0.5% E307])

Figure 7c shows that the modification with (SA + [1.5% K_3_ + 0.5% E307]) resulted in extensive stabilisation of this type of SAHL, while the depressed semicircle exhibited practically constant behaviour up to 7 days of immersion in the selected corrosion environment. The diameter of the depressed semicircle increased after one day of immersion. It did not increase to the same extent as with the modification of the Cu surface with (SA + 2.0% K_3_), it was only slightly lower, and, above all, remained constant. This can be interpreted as meaning that a lower proportion of the added K_3_ is available for direct and immediate binding to the copper surface which leads to a lower *R*_p_ during days 1–4 of immersion than in the case of (SA + 2.0% K_3_). The presence of the hydrophobic E307 also contributes to the increased stability of SAHL due to the hydrophobic interactions with the stearic acid preventing a deeper penetration of the corrosive medium into the protective layer. If we include the experimental results of the EIS measurements in the argumentation, we should first emphasise the constant increase in the film resistance value from 175 Ω cm^2^ (1 h) to 450 Ω cm^2^ (7 days) with a low or microscopic heterogeneity, which is confirmed by the initial high values of *n*_f_ = 0.998 (ideal performance) until the sixth day of immersion, then changing to 0.94. The capacitance of film *C*_f_ was constant, with slight fluctuations, on average around 0.02 μF cm^−2^. Regarding this value, there was no difference between the SAHL to which no E307 was added. The positive effect of the addition of E307 can be seen in the parameters relating to the properties of the pores. The *R*_po_ values showed an increasing trend from 0.11 MΩ cm^2^ (1 h) to 1.14 MΩ cm^2^ (after 7 days), which, given that the *R*_po_ remains constant and *n*_po_ fluctuates around 0.98, could be attributed to the effective formation of a more compact layer with high resistance to ion transport. The value of the *R*_ct_ increased from 9.0 MΩ cm^2^ to 13.5 MΩ cm^2^ after one hour of immersion and remained constant until the seventh day of immersion when it decreased to 12.4 MΩ cm^2^. The *R*_p_ was also high and, stabilised at 14.0 MΩ cm^2^ after one day of immersion. This can also be seen in the Bode plot (Figure 6c), which shows the total impedance in the low frequency range, i.e., at the lowest frequency of 1 mHz. It was 3.0 MΩ cm^2^ lower than in the case of the modification (SA + 2.0% K_3_), but remained constant, while, in the case of (SA + 2.0% K_3_) the degradation set in after 4 days and *R*_p_ decreased by almost a third. Thus, the stability of the SAHL was therefore achieved, and the results indicate that an experiment with increasing additions of E307 could bring an improvement. The next mixture for the modification of copper surface was (SA + [1.5% K_3_ + 0.5% E307]).

(SA + [1.0% K_3_+ 1.0% E307])

The Nyquist diagram, Figure 7d, where the surface has been modified with (SA + [1.0% K_3_ + 1.0% E307]), i.e., by the equal addition of both vitamins, shows three depressed semicircles clearly, indicating the complexity of the SAHL structure, and, at the same time, showing that the SAHL was structurally or functionally diverse, meaning that different processes contributed to the overall electrochemical reactivity of the system. If we now compare this SAHL (Figure 7d) with the SAHL from (SA + 2.0% E307) (Figure 7b), we can speak clearly of the synergistic effect of the two added vitamins. The size of the depressed semicircles, which also represent the values of *R*_p_ within the time window of 1 h to 7 days of immersion, were higher than in the case of (SA + 2.0% E307) and the maximum size of the depressed semicircle was practically reached when the sample was immersed for more than 1 day and remained constant, which predicts the stability of this type of SAHL Evidently, a 0.5% lower concentration of K_3_ than in the (SA + [1.5% K_3_ + 0.5% E307]) SAHL was sufficient to prevent possible anodisation of the copper, due to its binding affinity to the metal surface, and to increase the adhesion of the SAHL to the copper surface. At the same time, a 0.5% higher concentration of E307 increased the hydrophobicity of the SAHL by embedding itself in the hydrophobic areas created by the SA. This led to greater compactness of the SAHL and better corrosion resistance properties in the case of long-term exposure to the chosen corrosion medium. The following is an explanation of the functionality of SAHL with the composition (SA + [1.0% K_3_ + 1.0% E307]), based on the EIS measurements. In the high frequency range, *R*_f_ increased from 504 Ωcm^2^ to 692 Ωcm^2^ from 1 h immersion to seven days, the capacitance was constant and had the lowest values of all SAHLs i.e., *C*_f_ = 0.007 μFcm^−2^, as well as the parameter *n*_f_ = 0.99 up to the sixth day of immersion, after which it decreased minimally. The constant value of capacitance *C*_f_, which means that neither hydration nor degradation of the SAHL has taken place, indicates that the structure and thickness of the SAHL remained stable, which was reflected ultimately in the increase in resistivity *R*_f_. The middle frequency range also showed an increasing tendency of the pore resistance. The values of *R*_po_ increase continuously from 1.83 MΩ cm^2^ to 9.0 MΩ cm^2^ up to the seventh day of immersion, while the capacitance in this frequency range was also constant at around *C*_po_ = 0.01 μFcm^−2^, and the parameter *n*_po_ as well (*n*_po_ = 0.85). The increasing values of pore resistance and constant capacitance provide protection against electrolyte penetration into the pores, which means that the barrier function was maintained. This is due mainly to the higher concentration of E307, which allows the incorporation of E307 into the hydrophobic areas of the alkyl tails of the stearic acid. In the low-frequency range, the *R*_ct_ values increased from 12.0 MΩcm^2^ to 18.0 MΩ cm^2^ between the start of the measurement and the first day of immersion, rose significantly to 29.0 MΩcm^2^ by the fourth day and to 31.0 MΩcm^2^ on the sixth day, which represents the highest *R*_ct_ values of all the SAHLs in this period. This remarkable increase in charge transfer resistance can be explained by the complete stabilisation of the layer, which was also confirmed by the decrease the reduction of *C*_dl_ from 9 μFcm^−2^ to 3.0 μFcm^−2^ (narrowing of the pores). The polarisation resistance values *R*_p_ were correspondingly high, increasing from 13.6 MΩcm^2^ to 40.6 MΩcm^2^ over the days of exposure.

### 3.3. Surface Analyses of the SAHLs on Copper

#### 3.3.1. FTIR Analysis

**Figure 9:**
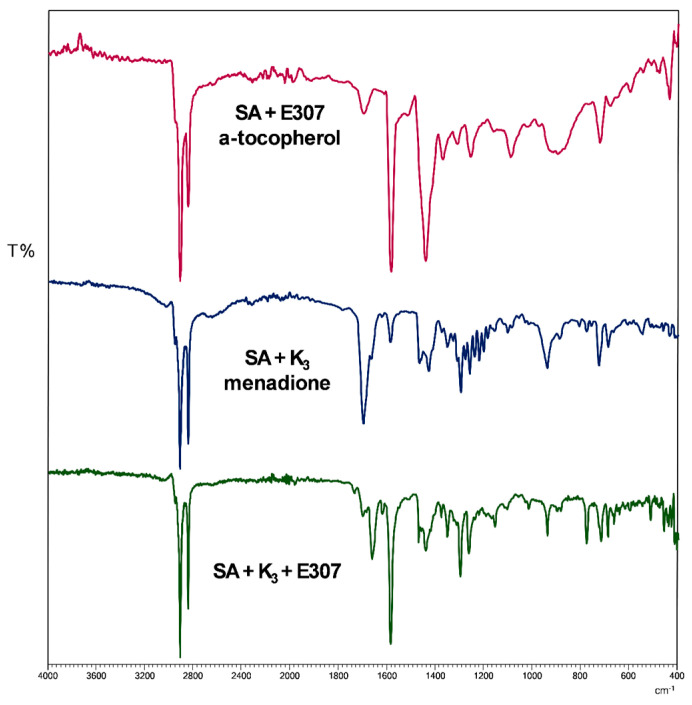
Structural formulae of individual components included in SAHLs and ATR-FTIR spectra of SAHLs on copper: (**top**) (SA + 2.0% E307), (**middle**) (SA + 2.0% K_3_), and (**bottom**) (SA + [1.0% K_3_ + 1.0% E307]), (transmittance vs. wavenumber).

The FTIR spectra of the SAHLs composed of stearic acid (SA) with α-tocopherol (E307), vitamin K_3_ (menadione) (SA + 2.0% E307), (SA + 2.0% K_3_) and their combination (SA + [1.0% K_3_ + 1.0% E307]) show characteristic vibrational features associated with both the stearic acid matrix and the incorporated molecules (Figure 9). Common to all three systems are strong CH_2_ stretching vibrations at about 2920 cm^−1^ (asymmetric) and 2850 cm^−1^ (symmetric), minor CH_2_ bending vibrations in the range of 1465–1375 cm^−1^, and common C–H bending and rocking modes of the alkyl chains in the fingerprint region (≈1500–600 cm^−1^), which contains characteristic vibrational patterns unique to specific molecular structures [60,62,63]. The low-intensity features near 500 cm^−1^ indicate weak Cu–O interactions at the metal–organic interface. These common spectroscopic signatures confirm that stearic acid formed a stable hydrophobic backbone in all three systems. In the SA + E307 system, a broad absorption band in the range of 3300–3400 cm^−1^ corresponds to the phenolic O-H stretch of α-tocopherol [73,74]. Its width and lower intensity indicate hydrogen bonding and embedding within the hydrophobic environment of the stearic acid chains. Further contributions in the fingerprint region, especially around 1160–1100 cm^−1^, can be assigned to the C-O stretching vibrations of the chromanol ring, while weak bands between 900 and 700 cm^−1^ correspond to the out-of-plane C-H bending modes of the ring [75]. For the SA + K_3_ system, a sharp carbonyl stretching vibration is observed at 1680–1700 cm^−1^, which can be assigned to the conjugated C=O group of the menadione. The shift relative to the free molecule indicates interactions either with the copper substrate or with neighbouring molecules. Aromatic C=C stretching bands appear between 1610 and 1450 cm^−1^, while in-plane C-H bending vibrations around 1200–1000 cm^−1^, and out-of-plane quinone vibrations at 900–800 cm^−1^ can be recognised in the fingerprint region [76]. In the ternary system (SA + [1.0% K_3_ + 1.0% E307]), the spectrum combines the diagnostic features of both vitamins. The broad O-H absorption of α-tocopherol around 3300 cm^−1^ coexists with the sharp C=O band of menadione at 1690 cm^−1^. In the fingerprint region, overlapping C-O stretching vibrations of tocopherol (≈1160–1100 cm^−1^) and in-plane C-H bending modes of quinone (1200–1000 cm^−1^) are observed, together with out-of-plane aromatic vibrations at 900–800 cm^−1^. The simultaneous observation of these diagnostic bands confirms that both antioxidants were incorporated into the hydrophobic stearic acid matrix and formed a composite protective layer on the copper. Overall, the FTIR analysis demonstrated that the common CH_2_-related vibrations and the low-frequency Cu-O features reflect the persistent stearic acid backbone and interfacial binding to copper, while the high-frequency O-H and C=O bands and the fingerprint region vibrations confirm the presence of α-tocopherol, menadione, or both. In the ternary system, the coexistence of these bands indicates the simultaneous incorporation of both antioxidants, suggesting molecular interactions that may improve the stability and protective properties of the film on copper.

#### 3.3.2. SEM-EDAX Analyses

An additional EDS analysis was performed, in view of the pronounced morphological differences between the individual SAHLs visible on the SEM images. The aim of this analysis was to determine the elemental composition of the SAHL at selected locations on the surface of the analysed metal substrate, and to evaluate possible local variations in the chemical composition. The SAHL of (SA + 2.0% K_3_) exhibited a pronounced rod-like morphology, with rod lengths of approximately 1.5 to 3.0 µm and diameters between 100 and 250 nm (Figure 2a). These structures indicate oriented crystal growth, but the overall morphology appears to be less compact and more porous, with loosely organised bundles. While this architecture initially leads to high polarisation resistance, the porous nature may allow gradual penetration of the electrolyte, leading to decreasing resistance over several days of immersion, as confirmed by EIS. The EDS (Figure S1) showed a high carbon content (C = 94.0–96.0 at%), low oxygen (O = 3.5–5.3 at%) and traces of copper (Cu = 0.2–2.2 at%), suggesting a predominantly organic composition with occasional exposure of the substrate in thinner areas. The (SA + E307) layer had a smoother and more uniform surface, with less microstructural complexity and no rod-like or hierarchical features (Figure 2b). The initial polarisation resistance was lower, but the SAHL became protective over time, probably due to the antioxidant stabilising effect of E307, which improves structural integrity and prevents oxidative degradation. This leads to a significant increase in resistance after 2 days. The EDS (Figure S2) confirmed the predominance of carbon (C = 93.0–94.0 at%), with a very low oxygen content (≈1.9 at%) and a higher copper signal (Cu = 3.0 –6.0 at%). The most structurally sophisticated coating (SA+ [1.0% K_3_ + 1.0% E307]), showed the formation of flower-like hierarchical structures, with the microscale “flower” arrangements consisting of nanoscale platelets or petals (Figure 2c). This hierarchical architecture combined the directional crystal growth of K_3_ with the stabilising effect of vitamin E307, resulting in a high initial polarisation stability that remained almost constant over 5–6 days. The complex multi-level morphology probably provided both physical barrier properties and chemical resistance, which explains the superior long-term electrochemical stability. Three different compositional regimes (Figures S3 and S4) can be distinguished for SAHL: C = 90.0–92.0 at. %, O = 7.2–8.2 at. %, Cu = 0.8–0.9 at. %, then C = 90.0–91.0 at. %, O = 6.1–7.6 at. %, Cu = 2.3–2.5 at. %, or alternatively C = 94.0–95.0 at. %, O = 3.4–4.3 at. % and Cu = 1.1–2.6 at.%. It is important to note that the pronounced oxygen enrichment cannot be attributed solely to the oxygen-containing functional groups of SA and K_3_, since the binary (SA + 2.0% K_3_) layers contained on average only 4.0 at. % O. The additional increase to 6.0–8.0 at. % O therefore indicates that the E307 contributed significantly to the oxygen incorporation. Due to its own oxygen-rich functional groups and its interaction with the substrate, E307 promotes both increased retention of oxygen within the layer and local oxidation, resulting in chemically more heterogeneous areas. In contrast, the areas where a higher Cu content was detected most likely correspond to thinner or more porous parts of the coating where the analytical signal contains contributions from the metallic substrate.

The coexistence of O- and Cu-enriched regions thus reflects the combined effect of additively generated functional groups and the interaction with the substrate, which, together, determine the chemical heterogeneity and hierarchical growth of the plate-like structures that resemble flower-like structures [77].

## 4. Conclusions

To summarise, all investigated SAHLs formed on copper surface with (SA + 2.0 wt% K_3_), (SA + 2.0 wt% E307), as well as in the mixtures with a constant total additive content of 2.0 wt%: (SA + [1.5 wt% K_3_ + 0.5 wt% E307]) and (SA + [1.0 wt% K_3_ + 1.0 wt% E307]) exhibited high inhibition efficiency (99%), which remained stable even during prolonged exposure. However, the EIS results indicated certain vulnerabilities, particularly for the modified copper surface with (SA + 2.0 wt% K_3_). Although this layer provided excellent initial protection, after four days it showed a noticeable decrease in performance, as reflected by a slightly reduced polarisation resistance. Considering that the SAHL with (SA + 2.0 wt% K_3_) exhibited significantly lower pore resistance compared to the SAHL with (SA + 2.0 wt% E307), it is reasonable to assume that the pores facilitate electrolyte penetration, indicating a deficiency in the hydrophobicity of this layer. By combining both vitamins, this drawback was effectively mitigated, resulting in improved barrier properties and enhanced overall stability of the SAHL. Modifying the copper surface with a mixture of both vitamins produced a synergistic effect, combining the properties exhibited by each vitamin when present individually in the layer. It should be emphasised that the polarisation resistance of the (SA + 2.0% E307) modified copper surface in the last two days after immersion in the corrosive medium was approximately the same as for (SA+ [1.5% K_3_ + 0.5% E307]), while in a time window of 1 to 6 days, it deviated by more than 50% in favour of (SA+ [1.0% K_3_ + 1.0% E307]).

## Figures and Tables

**Table 1:** Kinetic parameters for the corrosion of Cu obtained from potentiodynamic polarisation curves for the bare and modified surfaces of the simulated acidic urban rain solutions (pH = 5) at 25 °C (long-term test); A—bare surface, B—modified surface with (SA + 2.0% K_3_), C—modified surface with (SA + 2.0% E307), D—modified surface with (SA + [1.5% K_3_ + 0.5% E307]), E—modified surface with (SA + [1.0% K_3_ + 1.0% E307]).

	Time (Day)	*i*_corr_ (nAcm^−2^)	*E*_corr_ (V/SCE)	*R*_p_ (MΩcm^2^)	*b*_c_ (mV/dec)	*b*_a_ (mV/dec)	% *ηi*_corr_	*% ηR*_p_
A		1731.76	−0.044	0.10	290.03	81.60		
	1 d	0.10	−0.028	19.721	61.62	41.65	99.99	99.55
B	4 d	1.00	−0.045	16.416	75.92	86.93	99.94	99.47
	6 d	31.72	−0.019	9.162	163.75	59.27	98.17	99.05
	7 d	43.82	−0.011	4.330	223.15	40.26	97.47	97.99
	1 d	2.99	−0.078	7.481	131.86	153.11	99.83	98.84
C	4 d	3.84	−0.051	9.171	123.04	88.83	99.78	99.05
	6 d	6.75	−0.018	34.146	171.14	56.51	99.61	99.74
	7 d	8.45	−0.013	35.006	179.62	46.26	99.51	99.75
D	6 d	4.94	−0.069	13.573	138.61	41.52	99.71	99.36
E	6 d	2.47	−0.162	34.481	149.27	62.33	99.86	99.75

**Table 2:** Electrochemical impedance parameters (EIS fitting results) for copper (for the bare and modified surfaces) after immersion in simulated solution of acid rain with pH = 5 for different times at 25 °C; A—bare surface, B—modified surface with (SA + 2.0% K_3_), C—modified surface with (SA + 2.0% E307), D—modified surface with (SA + [1.5% K_3_ + 0.5% E307]), E—modified surface with (SA + [1.0% K_3_ + 1.0% E307]).

	Time (Hour) (Day)	*R*_s_ (kΩcm^2^)	*R*_f_ (kΩcm^2^)	*n*_f_	*C*_f_ (nFcm^−2^)	*R*_po_ (MΩcm^2^)	*n*_po_	*C*_po_ (nFcm^−2^)	*R*_ct_ (MΩcm^2^)	*n*_ct_	*C*_dl_ (μFcm^−2^)	*R*p-EIS (MΩcm^2^)	% *ηR*_p-EIS_
A	1 h	0.199	1.511	0.869	2672.00	/	/	/	0.067	0.493	3297.91	0.069	
	1 d	0.191	3.000	0.661	2306.00	/	/	/	0.080	0.478	999.85	0.083	
	4 d	0.175	3.665	0.680	1879.00	/	/	/	0.096	0.510	287.54	0.100	
	6 d	0.161	6.305	0.705	1550.00	/	/	/	0.103	0.569	63.71	0.109	
	7 d	0.152	8.821	0.706	1708.00	/	/	/	0.120	0.598	96.72	0.129	
B	1 h	0.199	0.205	0.999	9.40	0.367	0.946	57.20	7.068	0.528	6.66	7.435	99.08
	1 d	0.191	0.166	0.998	10.80	3.909	0.946	62.80	14.003	0.535	5.79	17.912	99.54
	4 d	0.175	0.165	0.999	10.90	4.003	0.946	63.20	13.474	0.513	5.60	17.477	99.43
	6 d	0.161	0.138	0.998	10.40	0.045	0.910	81.30	11.494	0.532	6.12	11.539	99.05
	7 d	0.152	0.137	0.999	10.90	0.035	0.921	97.10	6.486	0.435	7.92	6.521	98.03
C	1 h	0.497	1.705	0.982	9.40	6.791	0.997	40.30	2.313	0.596	6.87	9.104	99.25
	1 d	0.491	1.710	0.979	8.60	7.473	0.883	13.90	5.235	0.543	1.63	12.709	99.35
	4 d	0.395	0.801	0.948	8.30	8.346	0.997	21.50	10.437	0.505	2.75	18.783	99.47
	6 d	0.380	0.724	0.973	7.60	13.180	0.982	29.10	25.821	0.502	2.10	39.001	99.72
	7 d	0.359	0.579	0.962	8.90	10.244	0.983	31.30	31.481	0.591	1.09	41.725	99.69
D	1 h	0.304	0.175	0.999	10.10	0.107	0.840	22.70	9.039	0.428	9.47	9.146	99.25
	1 d	0.288	0.285	0.999	7.50	0.483	0.858	26.60	13.328	0.542	5.25	13.811	99.40
	4 d	0.260	0.399	0.999	8.40	0.771	0.879	13.90	13.549	0.490	4.37	14.317	99.30
	6 d	0.198	0.401	0.939	25.00	0.782	0.858	19.40	13.569	0.675	5.41	14.351	99.24
	7 d	0.171	0.451	0.939	36.80	1.140	0.857	18.80	12.419	0.578	2.66	13.559	99.05
	1 h	0.418	0.504	0.999	7.70	1.829	0.496	15.60	11.801	0.532	9.32	13.631	99.50
	1 d	0.388	0.566	0.999	7.60	5.776	0.682	13.20	18.234	0.473	7.94	24.011	99.66
E	4 d	0.376	0.602	0.991	5.50	7.576	0.680	8.90	29.217	0.495	5.16	36.794	99.73
	6 d	0.373	0.686	0.961	7.80	9.095	0.671	11.30	31.082	0.545	5.24	40.178	99.73
	7 d	0.300	0.692	0.930	7.70	8.815	0.586	57.20	31.099	0.511	3.05	40.606	99.68

## Data Availability

The original contributions presented in this study are included in the article/Supplementary Materials. Further inquiries can be directed to the author.

## Supplementary Materials

The following supporting information can be downloaded at: https://www.mdpi.com/article/10.3390/ma18225107/s1, Figure S1: EDAX (SA +2% K_3_), Figure S2: EDAX (SA +2% E307), Figure S3: EDAX (SA+ [1.0% K_3_ + 1.0% E307]), Figure S4: EDAX (SA+ [1.0% K_3_ + 1.0% E307]).
