# Supplementary figures and images for: Prolonged Protection of Copper in Acidic Media Through the Synergistic Effect of Fat-Soluble Vitamins
Source: Materials (Basel). 2025 Nov 10;18(22):5107. doi: 10.3390/ma18225107 (PMC12654228; doi:10.3390/ma18225107)

SA + 2%K<sub>3</sub>

Electron Image 3

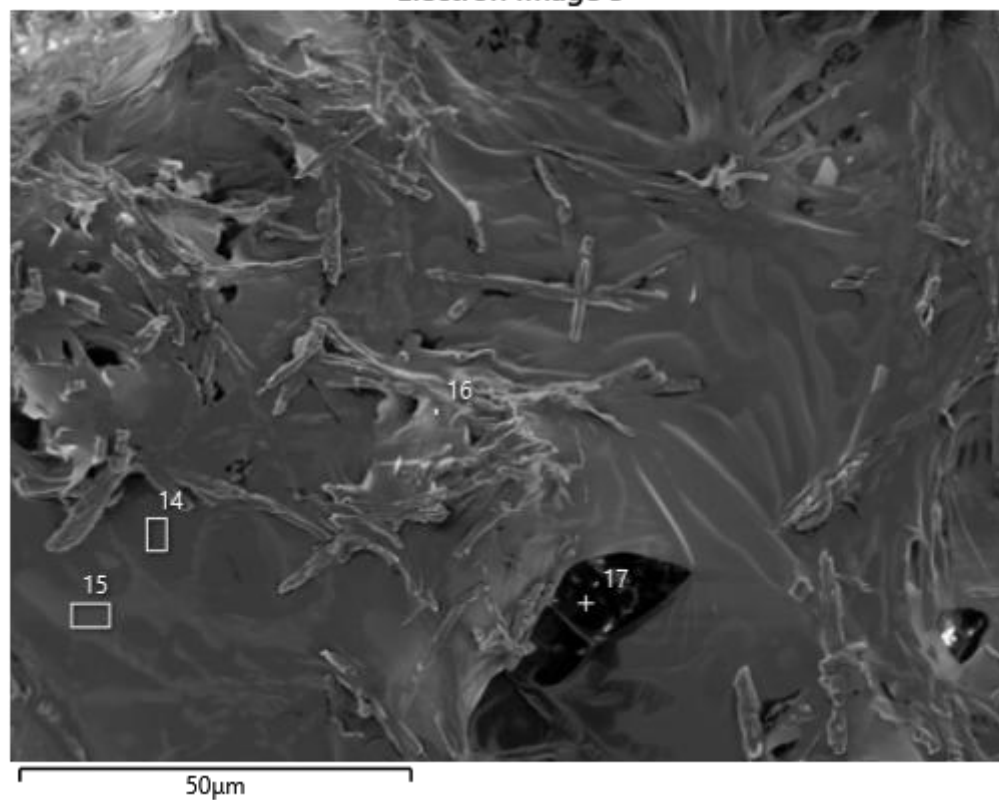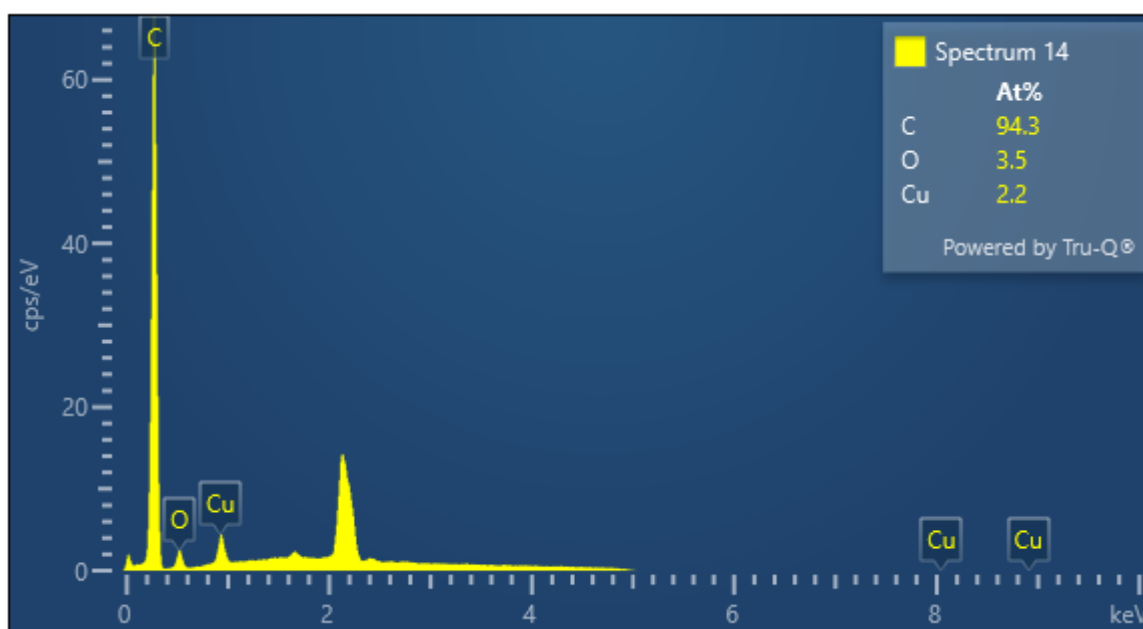

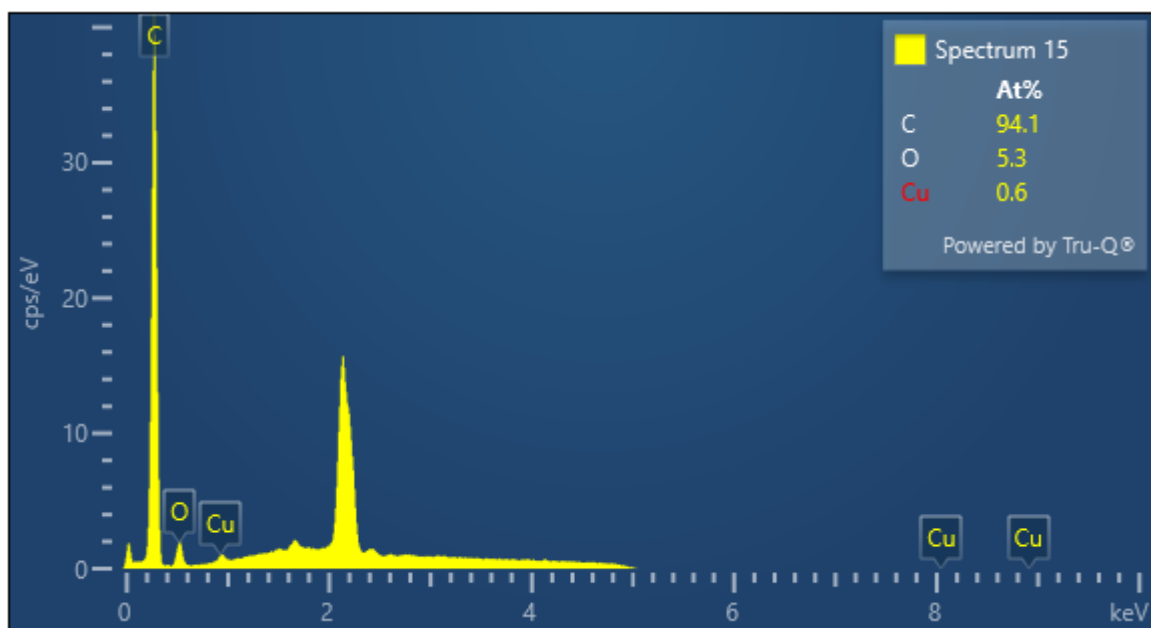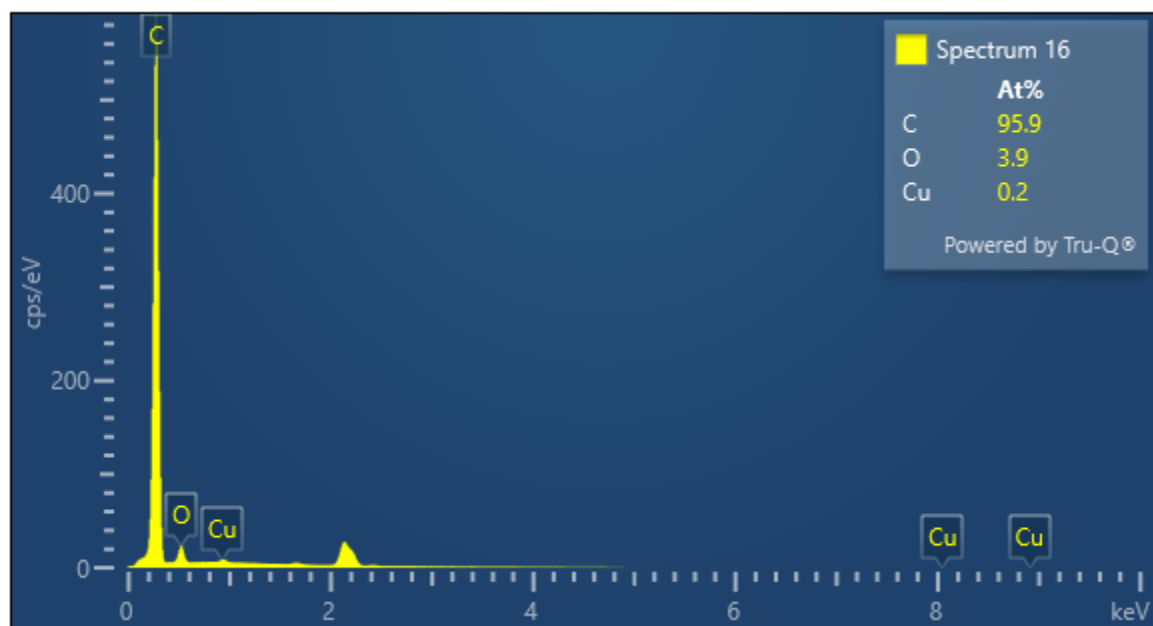

Supplement: Supplementary file 1 [file materials-18-05107-s001.zip › Figure S1 EDAX SA +2% K3.pdf]

SA + 2% E307

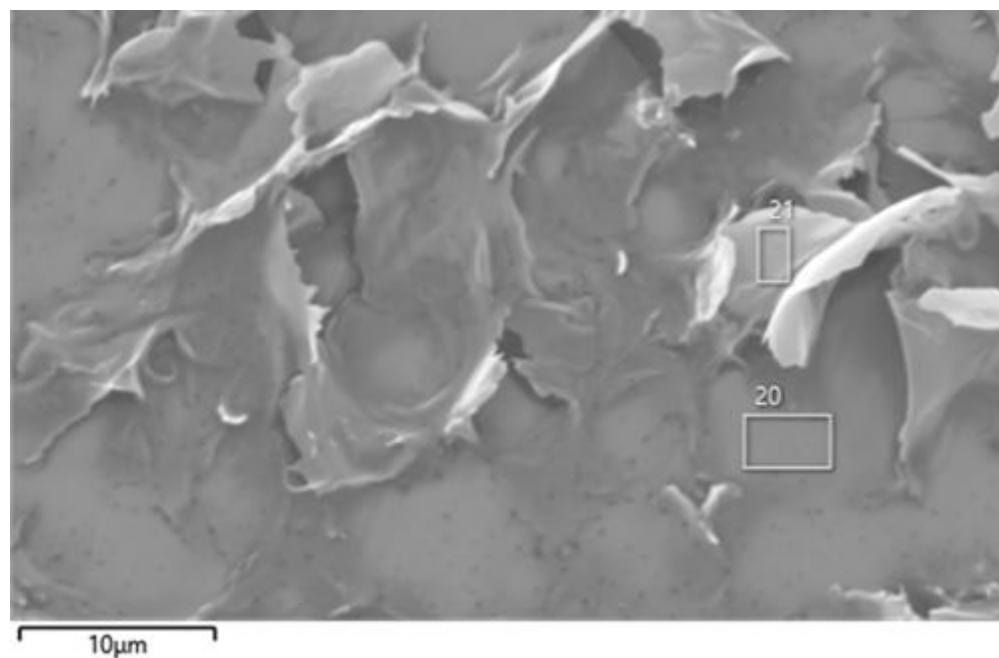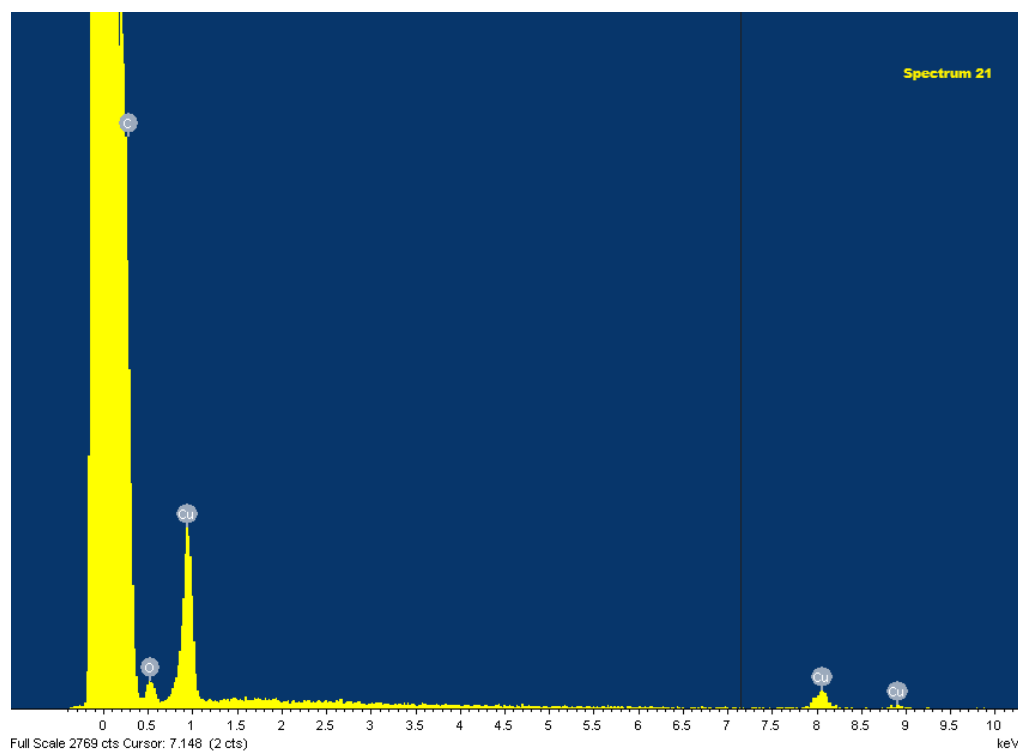

| Element | Weight% | Atomic% |
|---------|---------|---------|
| C K     | 78.65   | 93.76   |
| O K     | 2.14    | 1.91    |
| Cu L    | 19.22   | 4.33    |

|        |        |
|--------|--------|
| Totals | 100.00 |
|--------|--------|

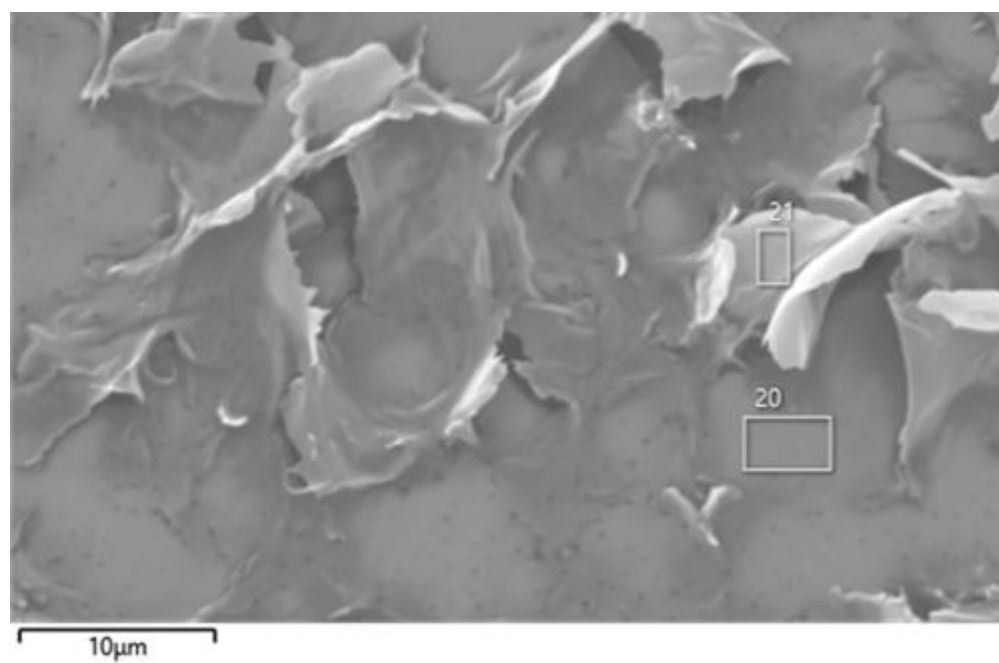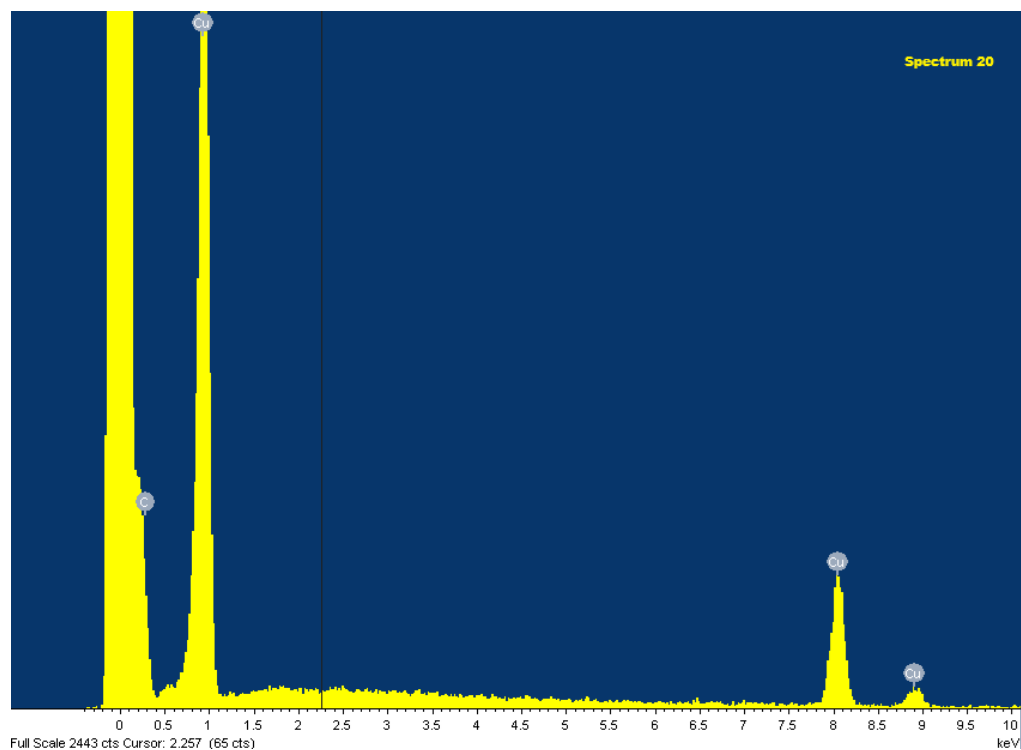

| Element | Weight% | Atomic% |
|---------|---------|---------|
| C K     | 79.87   | 94.75   |
| Cu L    | 14.20   | 6.25    |

---

|        |        |
|--------|--------|
|        |        |
| Totals | 100.00 |

---

Supplement: Supplementary file 1 [file materials-18-05107-s001.zip › Figure S2 EDAX SA +2% E307.pdf]

SA +(1% K3 + 1% E307)

Electron Image 1

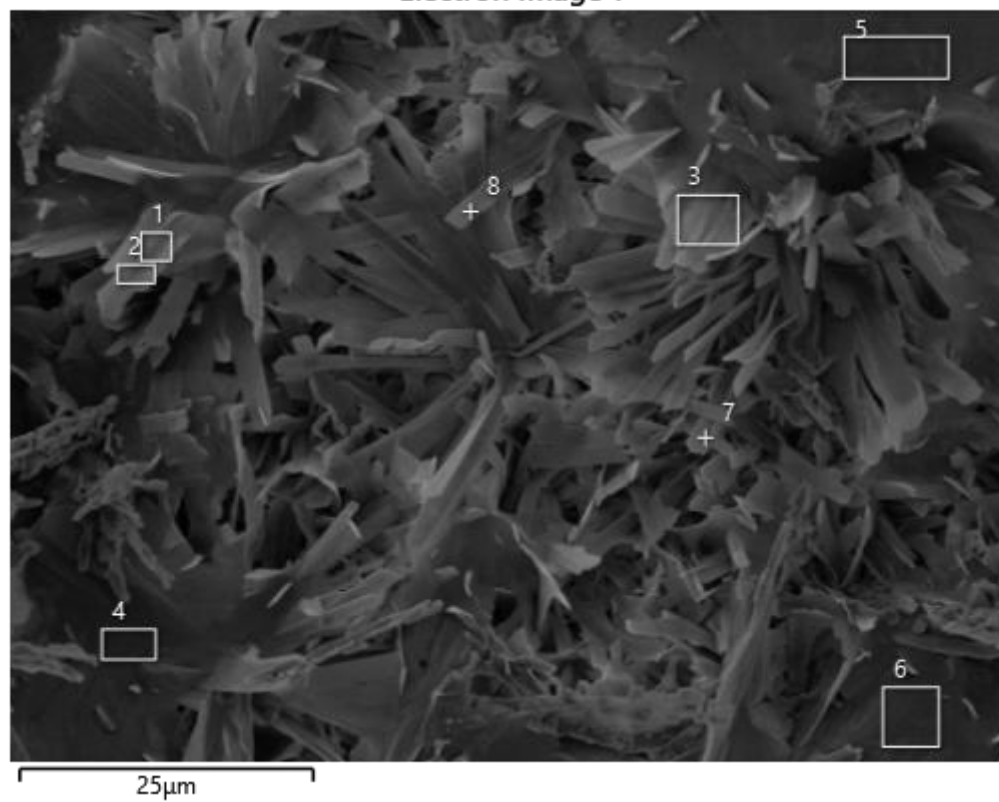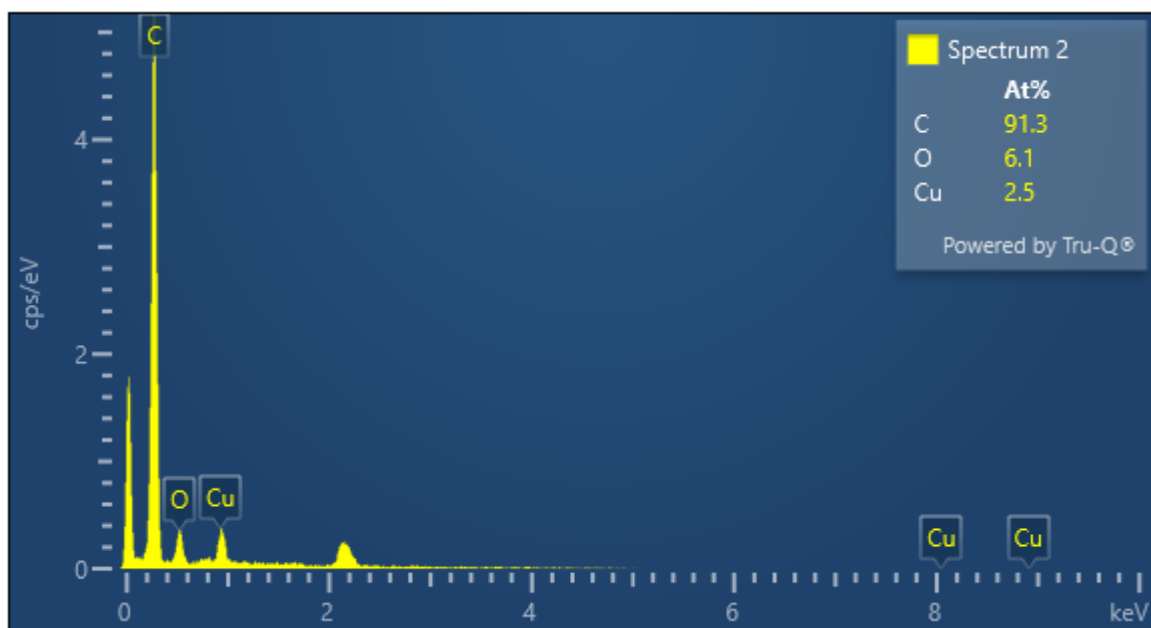

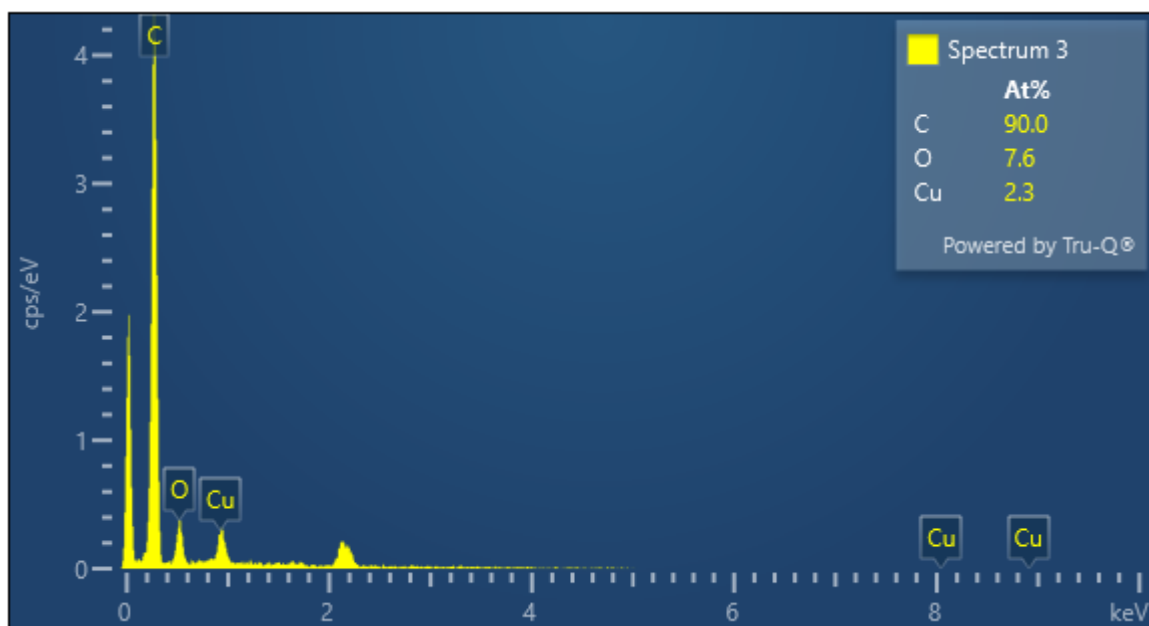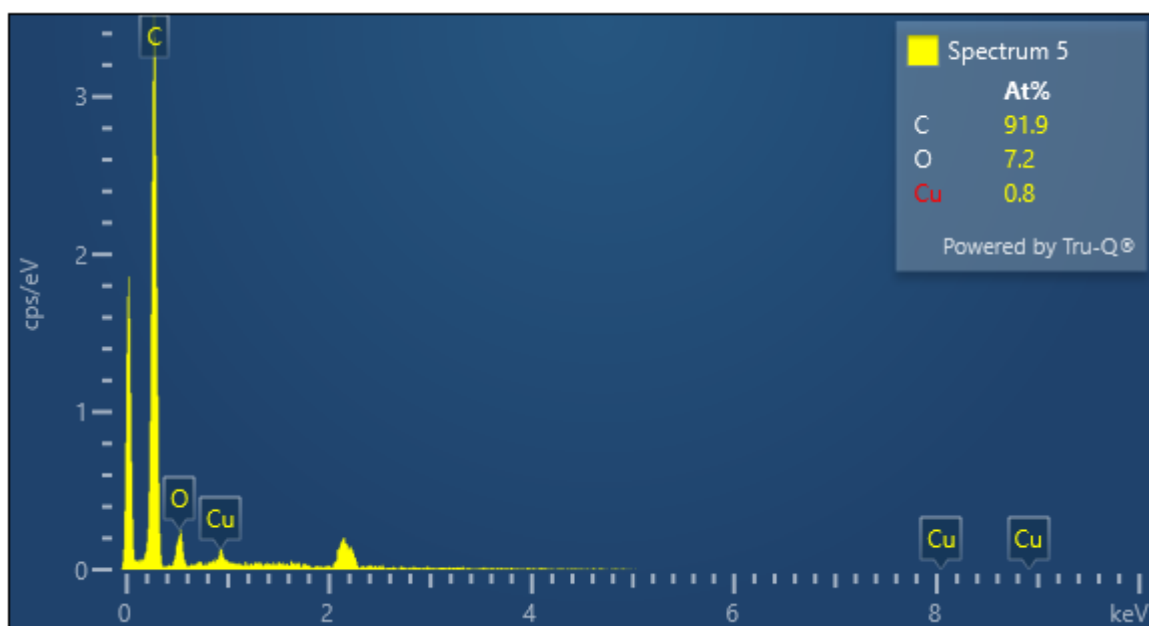

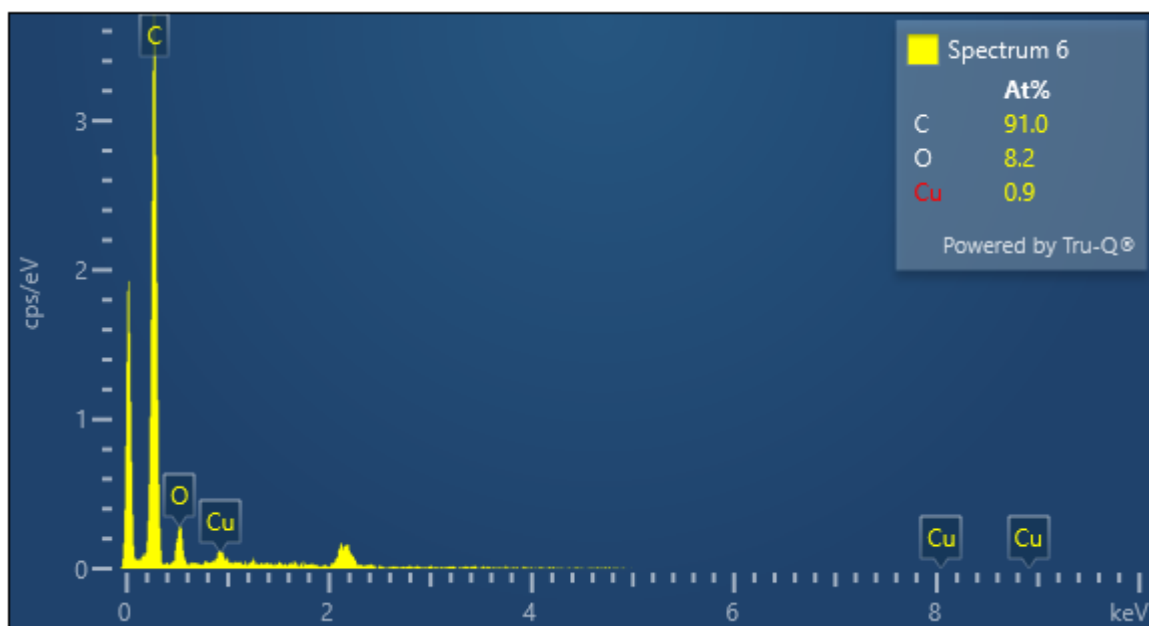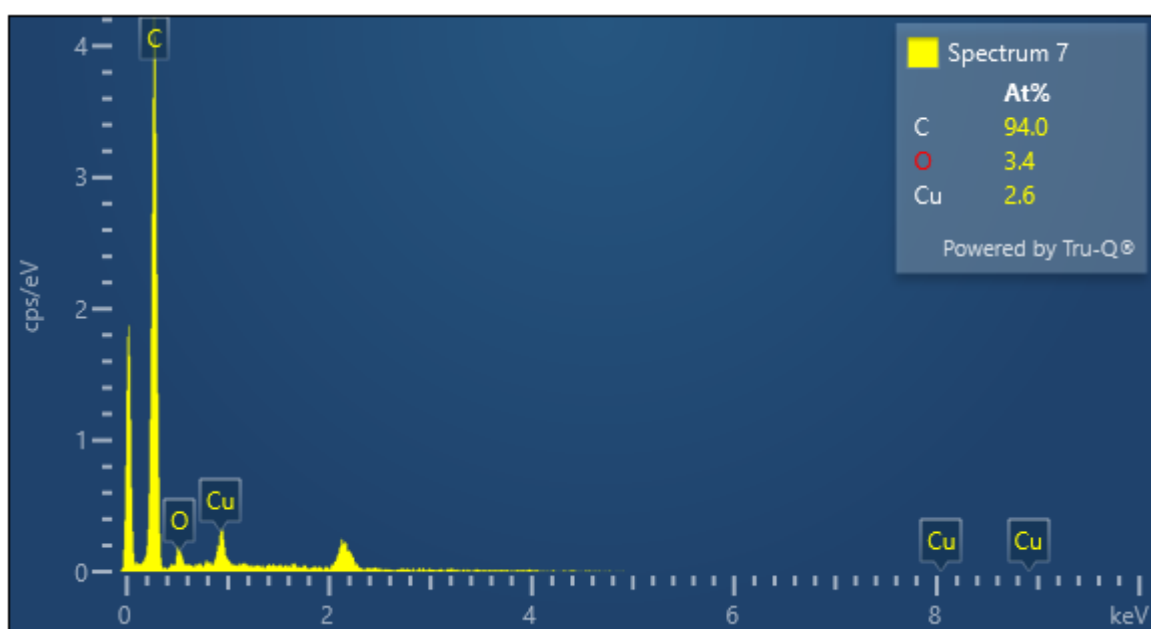

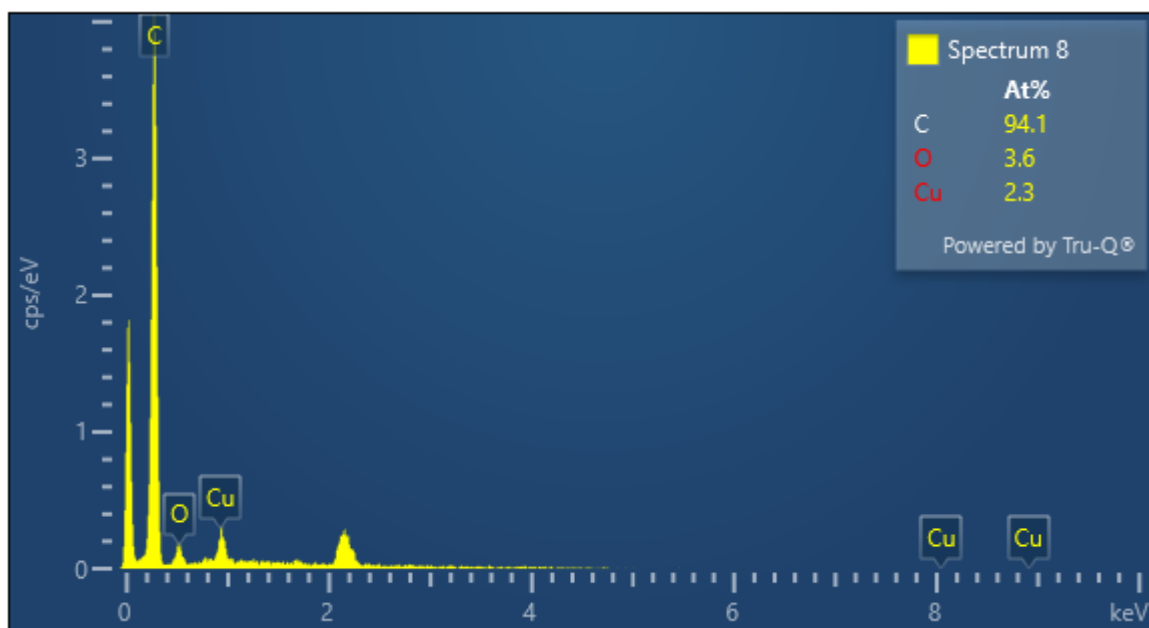

Supplement: Supplementary file 1 [file materials-18-05107-s001.zip › Figure S3 EDAX SA +(1% K3+1%E307).pdf]

SA +(1% K3 + 1% E307)

Electron Image 2

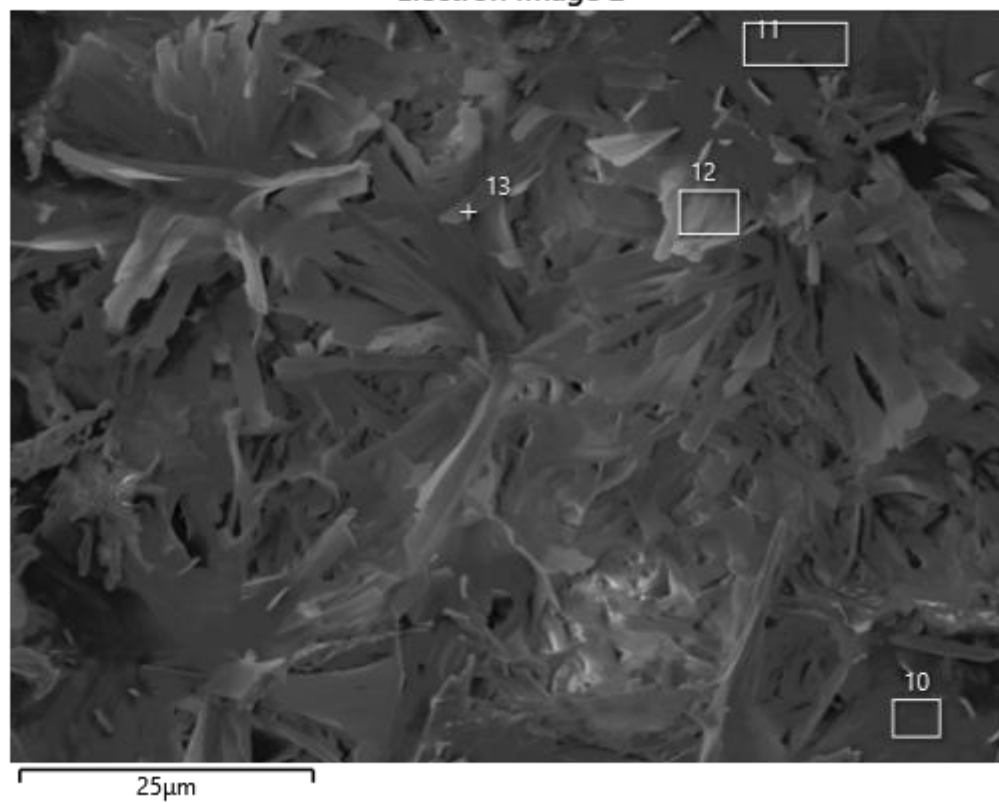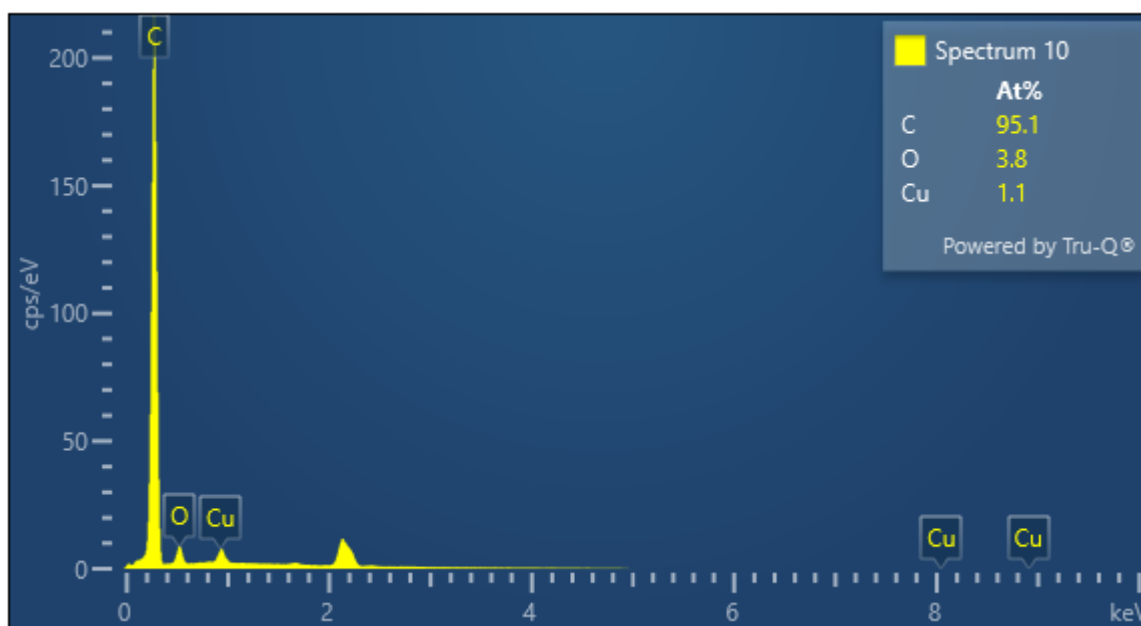

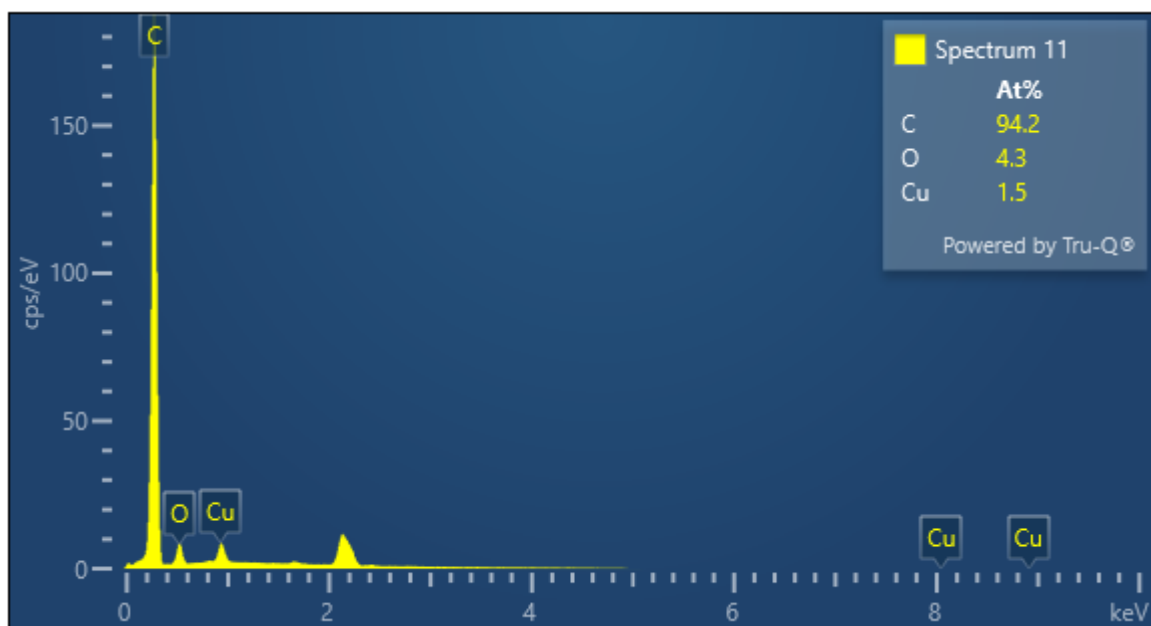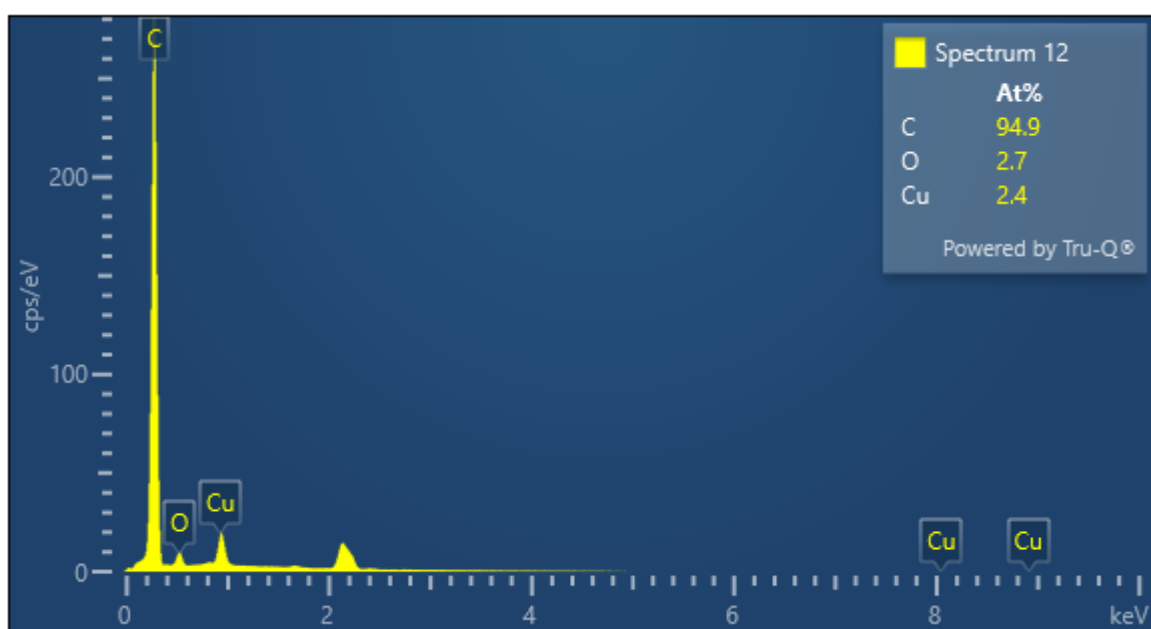

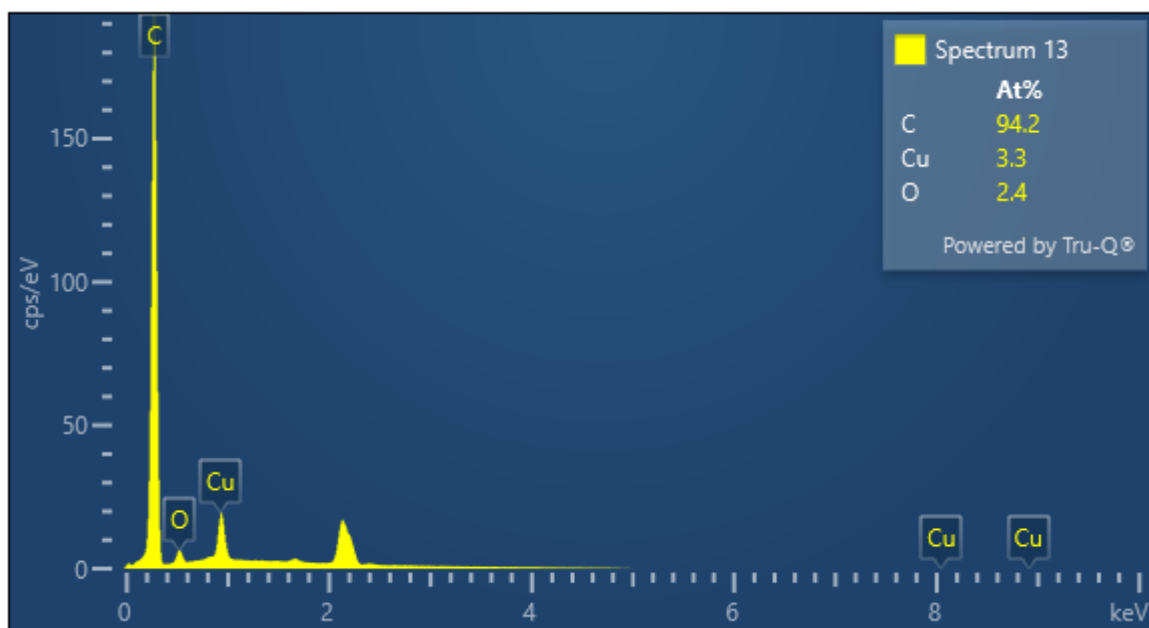

Supplement: Supplementary file 1 [file materials-18-05107-s001.zip › Figure S4 EDAX SA +(1% K3+1%E307).pdf]
